# Systematic Design and Synthesis of Methacrylate- and Trimethoxysilane-Functionalized POSS and DDSQ Nanostructures

**DOI:** 10.3390/nano16211343

**Published:** 2026-10-23

**Authors:** Bu-Hyun Moon, Eo Jin Oh, Hea Jung Park, Dong-Soo Shin

**Affiliations:** 1 R & D Center, Unis Co., Ltd., 1394, Nakdong-daero, Pusan 46907, Republic of Korea; mbhyun@naver.com; 2 Department of Chemistry, Changwon National University, Changwon 51140, Republic of Korea; eujin01.oh@gmail.com

**Keywords:** POSS, DDSQ, silsesquioxane, hydrosilylation, methacrylate functionalization, trimethoxysilane functionalization, organic–inorganic hybrid materials

## Abstract

Polyhedral oligomeric silsesquioxane (POSS) and double-decker silsesquioxane (DDSQ) are organic–inorganic hybrid nanostructures with well-defined siloxane frameworks that can be functionalized with various organic groups. In this study, a systematic series of six functional silsesquioxane derivatives was prepared using three different molecular architectures, POSS-8, DDSQ-4, and DDSQ-2. Three methacrylate-functionalized derivatives and three trimethoxysilane-functionalized derivatives were prepared from the corresponding Si–H-containing POSS and DDSQ precursors through hydrosilylation reactions. The target compounds were obtained in good yields of 81–91%, and their molecular structures were characterized by FT-IR, ^1^H and ^13^C NMR, ^29^Si NMR, and MALDI-TOF mass spectrometry. The spectroscopic results confirmed the successful introduction of the methacrylate and trimethoxysilane groups while maintaining the characteristic silsesquioxane frameworks. The parallel functionalization of POSS-8, DDSQ-4, and DDSQ-2 established a systematic series of silsesquioxane molecular precursors differing in framework architecture and peripheral functional-group density. The resulting derivatives contain either methacrylate or trimethoxysilane functionalities; however, their subsequent polymerization, hydrolysis/condensation, and material performance were not evaluated in the present study. Accordingly, these compounds are regarded as potential molecular precursors for further investigation.

## 1. Introduction

The Polyhedral oligomeric silsesquioxane (POSS) and double-decker silsesquioxane (DDSQ) have attracted considerable attention as organic–inorganic hybrid materials because of their well-defined siloxane frameworks and facile functionalization with various organic groups [1,2,3,4,5,6,7]. POSS generally consists of a nanosized Si–O cage surrounded by organic substituents and can be incorporated into polymeric or molecular systems through physical blending or covalent bonding. POSS derivatives can be prepared either through functionalization of incompletely condensed silsesquioxanes or by direct formation of fully condensed structures containing the desired organic substituents [8,9,10,11]. The peripheral functional groups strongly influence the reactivity, compatibility, and subsequent processing of POSS-based materials.

DDSQ compounds possess a double-decker siloxane framework and have also been widely investigated as molecular building blocks for organic–inorganic hybrid materials. In particular, Si–H-functionalized POSS and DDSQ derivatives can undergo hydrosilylation reactions with unsaturated organic compounds, providing an efficient approach for introducing a variety of peripheral functional groups [6,12,13,14,15,16,17,18,19,20,21,22,23,24,25,26,27]. The combination of a stable siloxane framework and reactive organic substituents enables the preparation of silsesquioxane derivatives with controlled molecular architectures and functionalities.

Among the various functional groups that can be introduced into silsesquioxane frameworks, methacrylate and trialkoxysilane groups are of particular interest because of their established chemical reactivities. Methacrylate-containing compounds have been widely employed in radical polymerization and cross-linking reactions, whereas trialkoxysilane groups can undergo hydrolysis and condensation to form Si–O–Si networks [28,29,30,31,32,33]. These established reaction pathways provide the chemical rationale for introducing methacrylate and trimethoxysilane functionalities into well-defined POSS and DDSQ frameworks. In the present study, however, the resulting compounds are considered potential molecular precursors, and their polymerization, hydrolysis/condensation, and resulting material properties were not experimentally evaluated.

Although various POSS- and DDSQ-based derivatives have been reported, most previous studies have focused on individual silsesquioxane frameworks or a single type of reactive functionality. A systematic preparation of POSS and DDSQ architectures bearing different numbers and arrangements of peripheral functional groups remains limited. Systematically organized molecular series spanning different POSS and DDSQ architectures and peripheral functional-group densities remain relatively limited. The parallel introduction of methacrylate and trimethoxysilane functionalities into POSS-8, DDSQ-4, and DDSQ-2 therefore provides a common synthetic framework for preparing structurally related silsesquioxane precursors with different numbers and spatial arrangements of reactive groups.

**Figure 1:**
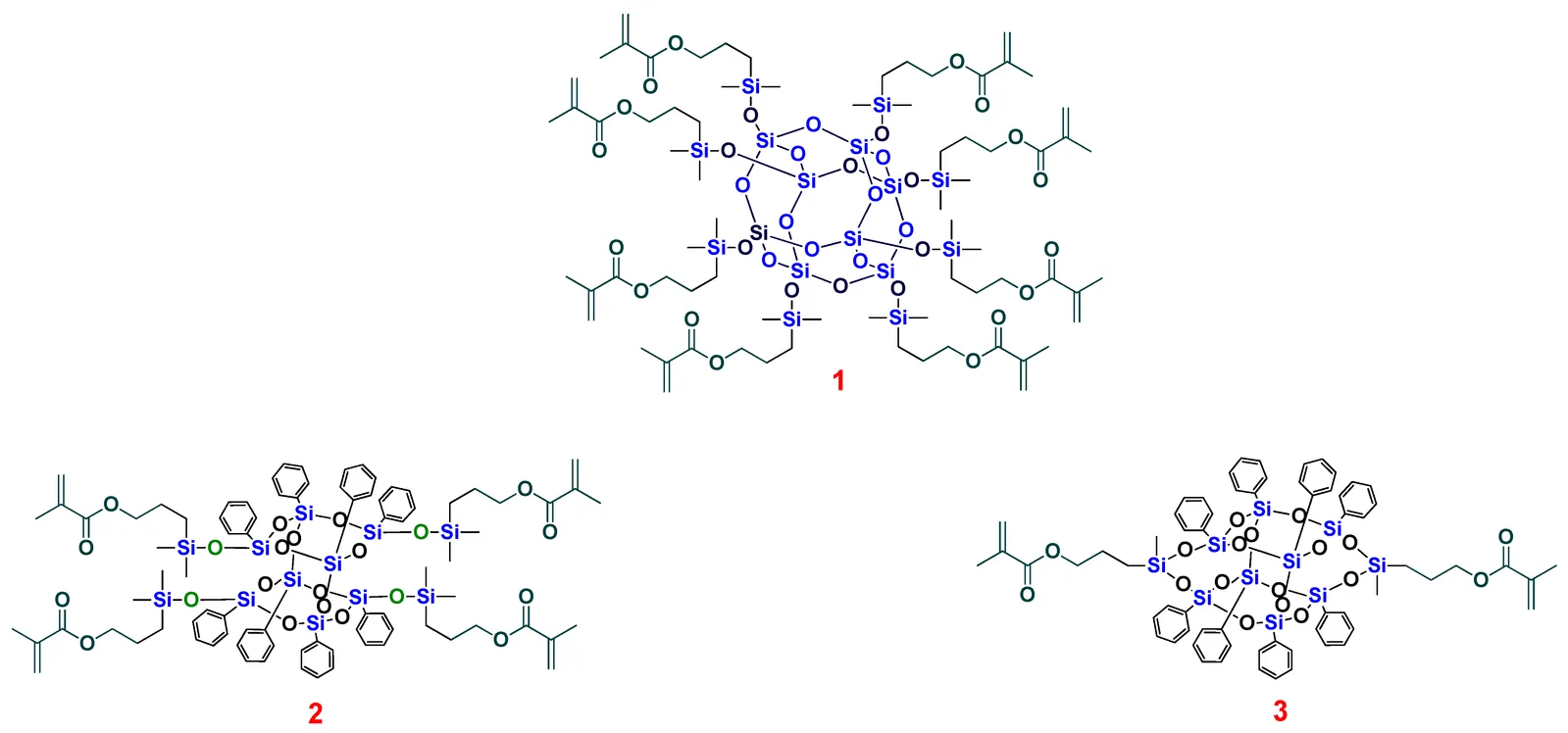
Chemical structures of POSS-8-O-Si-Methacryl (**1**), DDSQ-4-O-Si-Methacryl (**2**), and DDSQ-2-O-Si-Methacryl (**3**).

**Figure 2:**
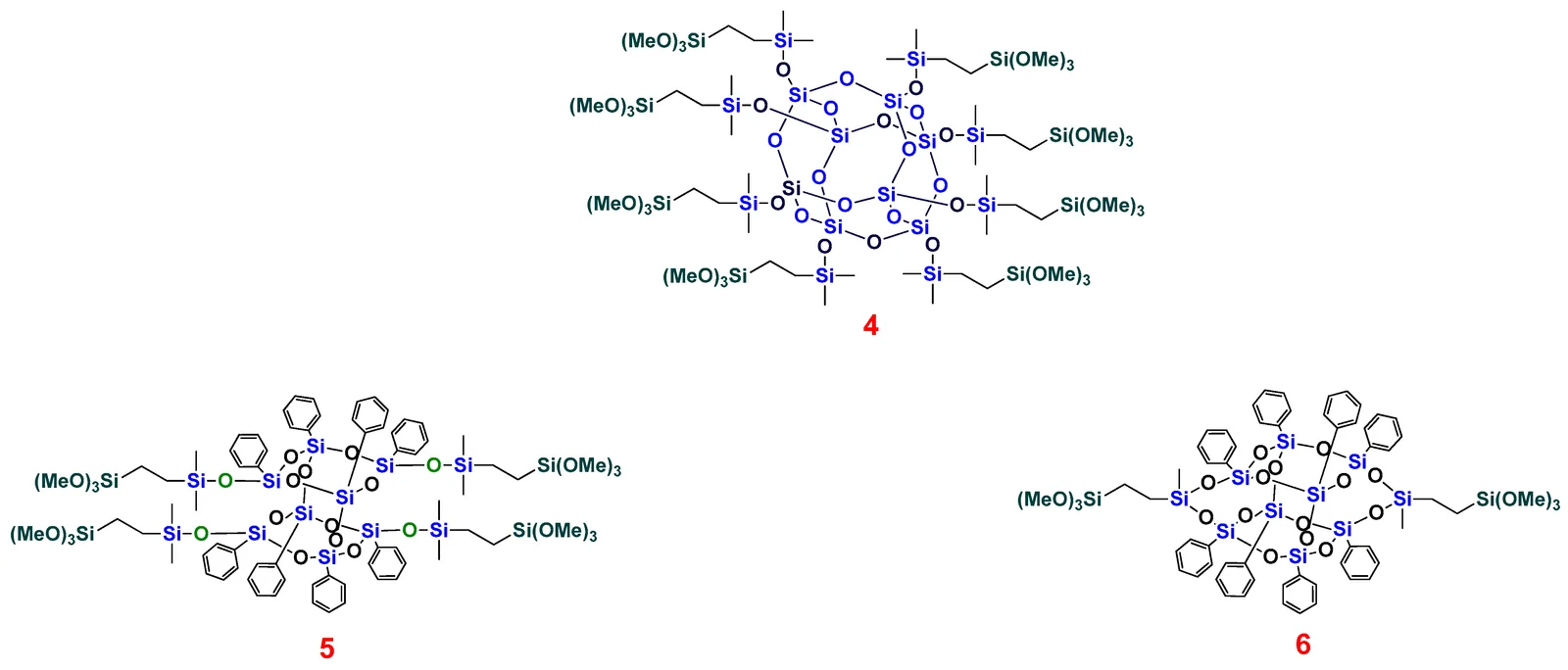
Chemical structures of POSS-8-O-Si-Trimethoxysilane (**4**), DDSQ-4-O-Si-Trimethoxysilane (**5**), and DDSQ-2-O-Si-Trimethoxysilane (**6**).

In this study, a systematic series of six methacrylate- and trimethoxysilane-functionalized silsesquioxane derivatives based on POSS-8, DDSQ-4, and DDSQ-2 was prepared using hydrosilylation reactions. (Figure 1 and Figure 2) Three methacrylate-functionalized derivatives, POSS-8-O-Si-Methacryl (**1**), DDSQ-4-O-Si-Methacryl (**2**), and DDSQ-2-O-Si-Methacryl (**3**), and three trimethoxysilane-functionalized derivatives, POSS-8-O-Si-Trimethoxysilane (**4**), DDSQ-4-O-Si-Trimethoxysilane (**5**), and DDSQ-2-O-Si-Trimethoxysilane (**6**), were prepared from the corresponding Si–H-functionalized precursors. Their molecular structures were investigated by FT-IR, ^1^H and ^13^C NMR, ^29^Si NMR, and MALDI-TOF mass spectrometry. The present work focuses on establishing and structurally characterizing this molecular precursor series rather than on comparing architecture-dependent reaction kinetics or application performance.

## 2. Materials and Methods

All commercially available starting materials, reagents, and solvents were purchased from commercial suppliers and used without further purification unless otherwise noted. Solvents were purchased from Daejung and dried according to the reported method. Column chromatography was performed on silica gel (60–120 mesh). Solvent evaporation was performed under reduced pressure using a rotary evaporator. NMR spectra were measured on a BRUKER AVANCE 400 (BRUKER, Rheinstetten, Germany) spectrometer in CDCl_3_ using residual low-purity solvent (CHCl_3_, ^1^H 7.26/^13^C 77.0) and tetramethylsilane (TMS, δ 0) as internal standards, and values are expressed in parts per million (ppm). IR spectra were recorded on an FT-IR-6300 (JASCO, Tokyo, Japan) spectrometer. Detailed synthetic procedures and characterization data are provided in the Supporting Information. MALDI-TOF mass spectra were acquired in positive-ion mode using super-DHB as the matrix and sodium acetate as the cationization agent. Samples were dissolved in THF at 5 mg mL^−1^ and mixed with the matrix and sodium acetate solution at a volume ratio of 50:50:1; therefore, molecular ions were evaluated as sodium adducts ([M+Na]^+^).

## 3. Results

### 3.1. Synthesis of POSS- and DDSQ-Based Derivatives

**Scheme 1:**
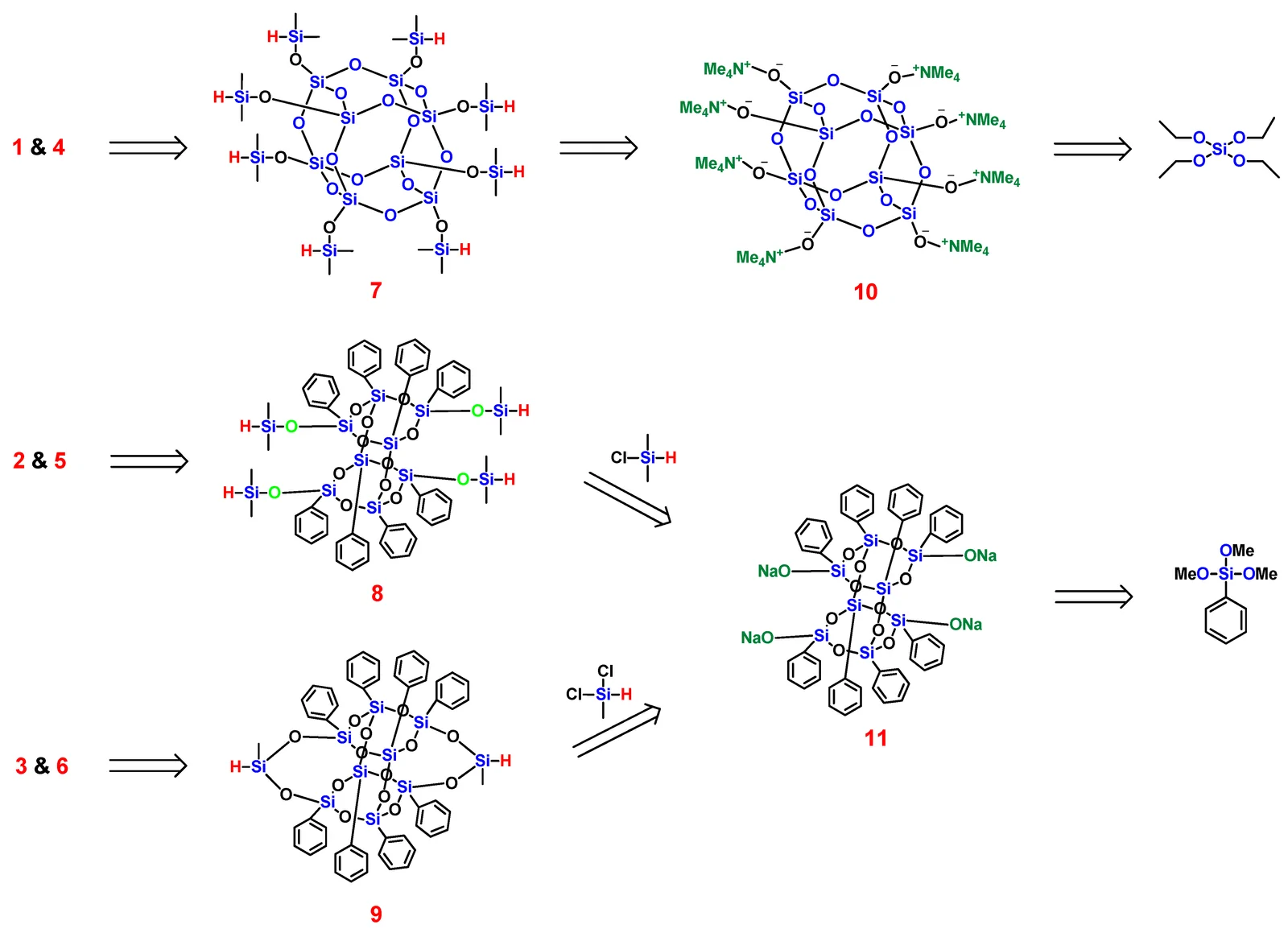
Retrosynthetic overview of the key intermediates for the preparation of POSS-8-O-Si-Methacryl (**1**), DDSQ-4-O-Si-Methacryl (**2**), DDSQ-2-O-Si-Methacryl (**3**), POSS-8-O-Si-Trimethoxysilane (**4**), DDSQ-4-O-Si-Trimethoxysilane (**5**), and DDSQ-2-O-Si-Trimethoxysilane (**6**). (Detailed synthetic routes and reaction conditions are shown in Scheme 2, Scheme 3 and Scheme 4.).

**Scheme 2:**
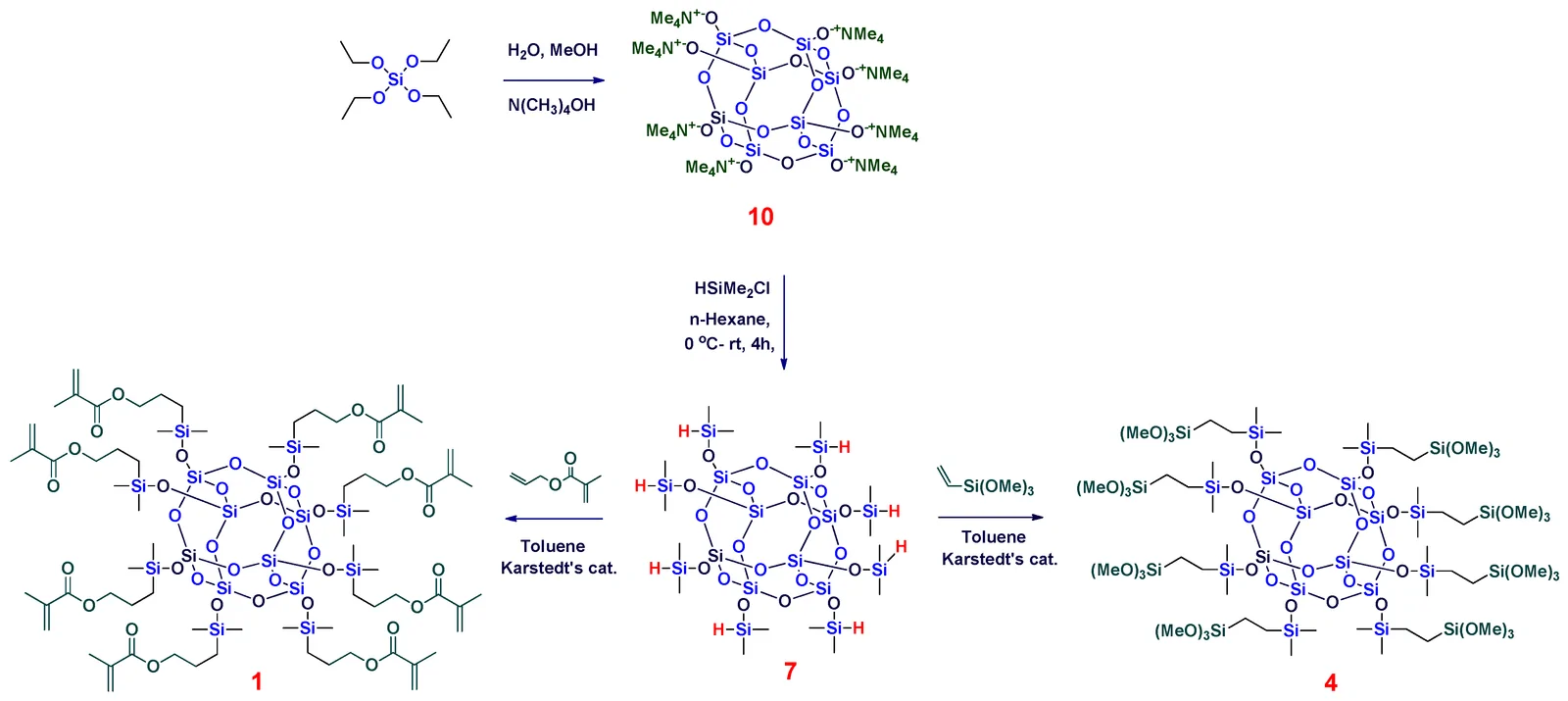
Synthesis of POSS-8-O-Si-Methacryl (**1**) and POSS-8-O-Si-Trimethoxysilane (**4**).

**Scheme 3:**
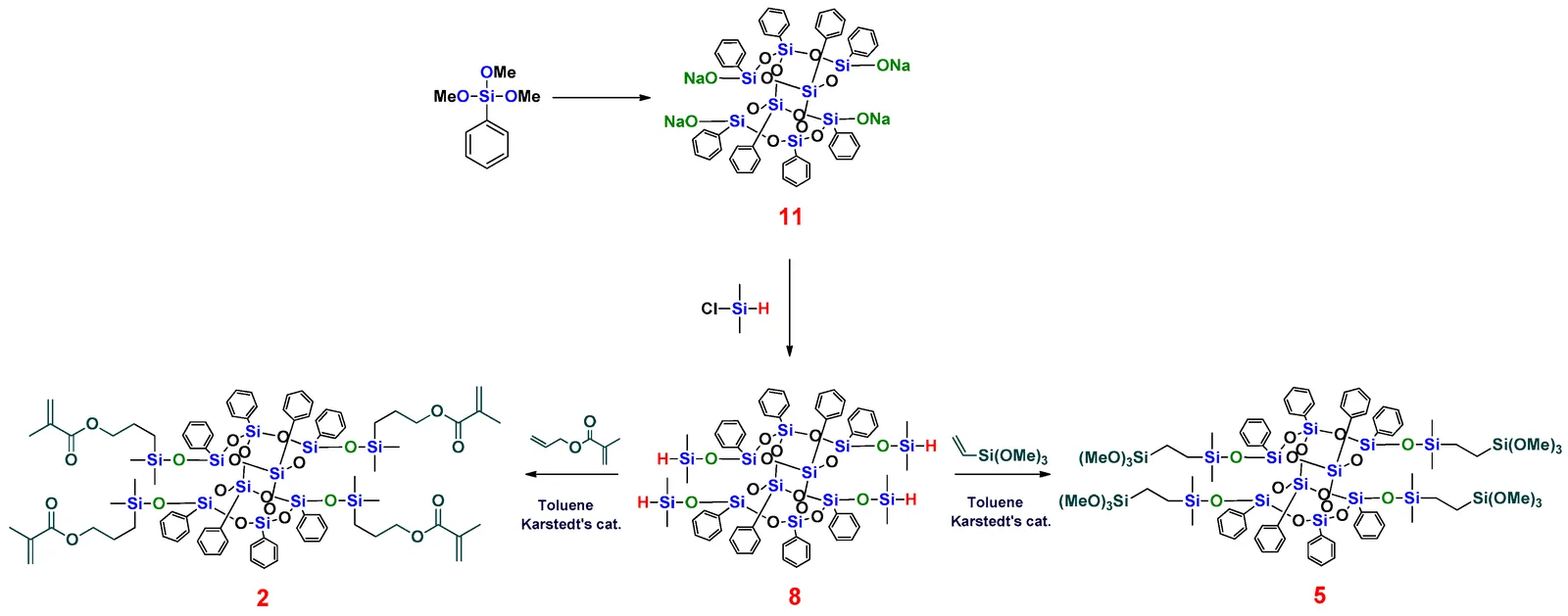
Synthesis of DDSQ-4-O-Si-Methacryl (**2**) and DDSQ-4-O-Si-Trimethoxysilane (**5**).

**Scheme 4:**
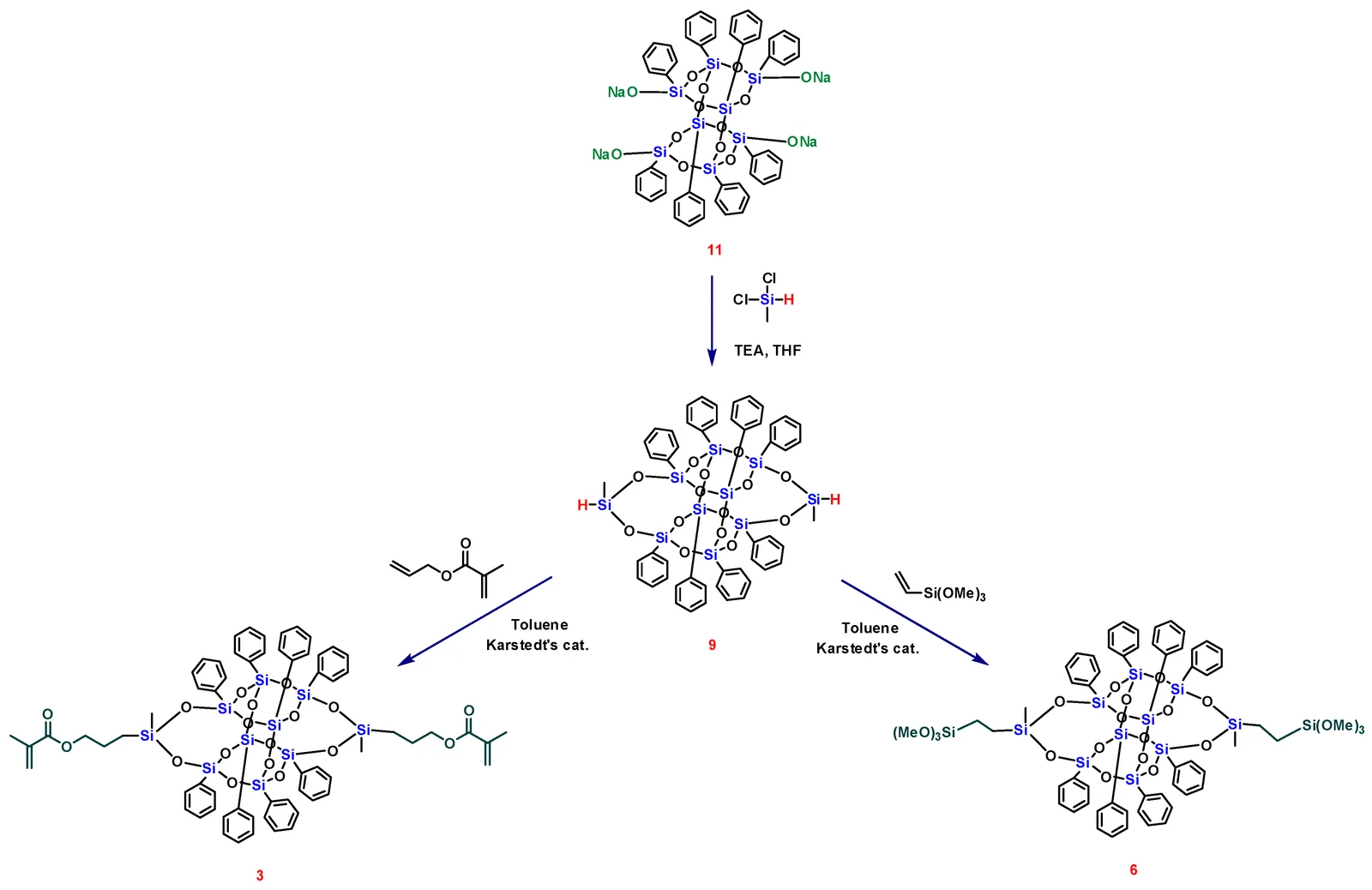
Synthesis of DDSQ-2-O-Si-Methacryl (**3**) and DDSQ-2-O-Si-Trimethoxysilane (**6**).

To establish a systematic functionalization strategy for structurally different POSS and DDSQ frameworks, three Si–H-functionalized silsesquioxane intermediates, POSS-8-O-Si-H (**7**), DDSQ-4-O-Si-H (**8**), and DDSQ-2-O-Si-H (**9**), were first synthesized. These intermediates contain different numbers and arrangements of reactive Si–H groups and were subsequently functionalized through hydrosilylation reactions to afford the corresponding methacrylate- and trimethoxysilane-containing derivatives. The overall retrosynthetic strategy is summarized in Scheme 1, while the detailed synthetic routes and reaction conditions are presented in Scheme 2, Scheme 3 and Scheme 4.

POSS-8-O-Si-H (**7**) was synthesized from tetraethoxysilane through a two-step reaction (Scheme 2). First, tetraethoxysilane was reacted with tetramethylammonium hydroxide in a MeOH/H_2_O mixture to give octakis(tetramethylammonium)-T8-silsesquioxane(10) [34]. The resulting silsesquioxane salt **10** was then reacted with dimethylchlorosilane in n-hexane to afford POSS-8-O-Si-H (**7**) in 91.8% yield. The Si–H groups of **7** were utilized for subsequent hydrosilylation reactions. Reaction of 7 with allyl methacrylate in the presence of Karstedt’s catalyst [35,36] afforded POSS-8-O-Si-Methacryl (**1**) in 81% yield. In a separate reaction, hydrosilylation of **7** with vinyltrimethoxysilane gave POSS-8-O-Si-Trimethoxysilane (**4**) in 85% yield. Thus, the Si–H-functionalized POSS framework could be efficiently converted into either a polymerizable methacrylate derivative or a trimethoxysilane-functionalized precursor.

The corresponding DDSQ derivatives were synthesized using DDSQ-4-ONa (**11**) as a common starting intermediate (Scheme 3 and Scheme 4). DDSQ-4-ONa (**11**) was prepared by reaction of phenyltrimethoxysilane with NaOH in an i-PrOH/H_2_O mixture. Reaction of **11** with chlorodimethylsilane afforded DDSQ-4-O-Si-H (**8**) in 93% yield. Hydrosilylation of **8** with allyl methacrylate (5 equiv.) in toluene at 60 °C using Karstedt’s catalyst (0.05 mL) gave DDSQ-4-O-Si-Methacryl (**2**) in 85% yield. In a separate reaction, hydrosilylation of **8** with vinyltrimethoxysilane (5 equiv.) in toluene at 70 °C using Karstedt’s catalyst (three drops) afforded DDSQ-4-O-Si-Trimethoxysilane (**5**) in 86% yield.

DDSQ-2-O-Si-H (**9**) was prepared by reacting DDSQ-4-ONa (**11**) with dichloro(methyl)silane and was obtained in 92% yield. Similar to the DDSQ-4 derivatives, the Si–H groups of **9** were subsequently subjected to hydrosilylation reactions. Hydrosilylation of **9** with allyl methacrylate (5 equiv.) in toluene at 60 °C using Karstedt’s catalyst (0.05 mL) afforded DDSQ-2-O-Si-Methacryl (**3**) in 87% yield. Reaction of **9** with vinyltrimethoxysilane (3 equiv.) in toluene at 70 °C using Karstedt’s catalyst (three drops) gave DDSQ-2-O-Si-Trimethoxysilane (**6**) in 91% yield.

Overall, the three Si–H-functionalized intermediates **7**–**9** were successfully converted into six methacrylate- and trimethoxysilane-functionalized POSS and DDSQ derivatives through hydrosilylation reactions. Application of the same general synthetic approach to the three structurally different silsesquioxane frameworks produced a systematic molecular series spanning different framework architectures and numbers and spatial arrangements of peripheral reactive groups. From a structural perspective, POSS-8 contains eight peripheral functional sites distributed around the compact T8 cage, whereas DDSQ-4 and DDSQ-2 contain four and two peripheral functional sites, respectively, on the double-decker framework. Thus, the present series provides a stepwise variation in both functional-group density and spatial arrangement. These structural differences may result in different steric environments around the peripheral sites; however, the present data do not permit a quantitative correlation between steric accessibility and intrinsic reactivity. Although all three architectures underwent hydrosilylation successfully, the present data do not allow a quantitative comparison of their intrinsic reaction kinetics because the reactions were not performed under strictly identical conditions. Time-resolved studies under standardized conditions would be required to determine whether POSS/DDSQ architecture influences hydrosilylation kinetics. Accordingly, the present study is limited to the preparation and structural characterization of these functional silsesquioxane precursors.

### 3.2. Structural Characterization of POSS- and DDSQ-Based Derivatives

The molecular structures of the synthesized methacrylate- and trimethoxysilane-functionalized POSS and DDSQ derivatives were investigated by FT-IR, ^1^H- and ^13^C-NMR, and ^29^Si-NMR spectroscopies. The characteristic spectral features of the methacrylate derivatives **1**–**3** and trimethoxysilane derivatives **4**–**6** were compared to confirm the introduction of the corresponding functional groups and the retention of the POSS and DDSQ frameworks. In the ^1^H NMR spectra of products **1**–**6**, the characteristic Si–H resonances of the corresponding precursors **7**–**9** were no longer observed. In addition, the integral ratios of the methacrylate or trimethoxysilane proton signals were consistent with the expected numbers of substituents, supporting full substitution of the available Si–H sites within the detection limits of the NMR measurements. The complete ^1^H NMR spectra of compounds **1**–**6** are provided in the Supporting Information (Figures S1–S6), allowing direct inspection of the Si–H region and the corresponding proton integrations. The room-temperature solution NMR data support the assigned molecular structures but do not exclude possible conformational dynamics or aggregation. These aspects were not investigated in the present study, and no conclusions regarding conformational behavior or aggregation are drawn from the present spectroscopic data.

**Figure 3:**
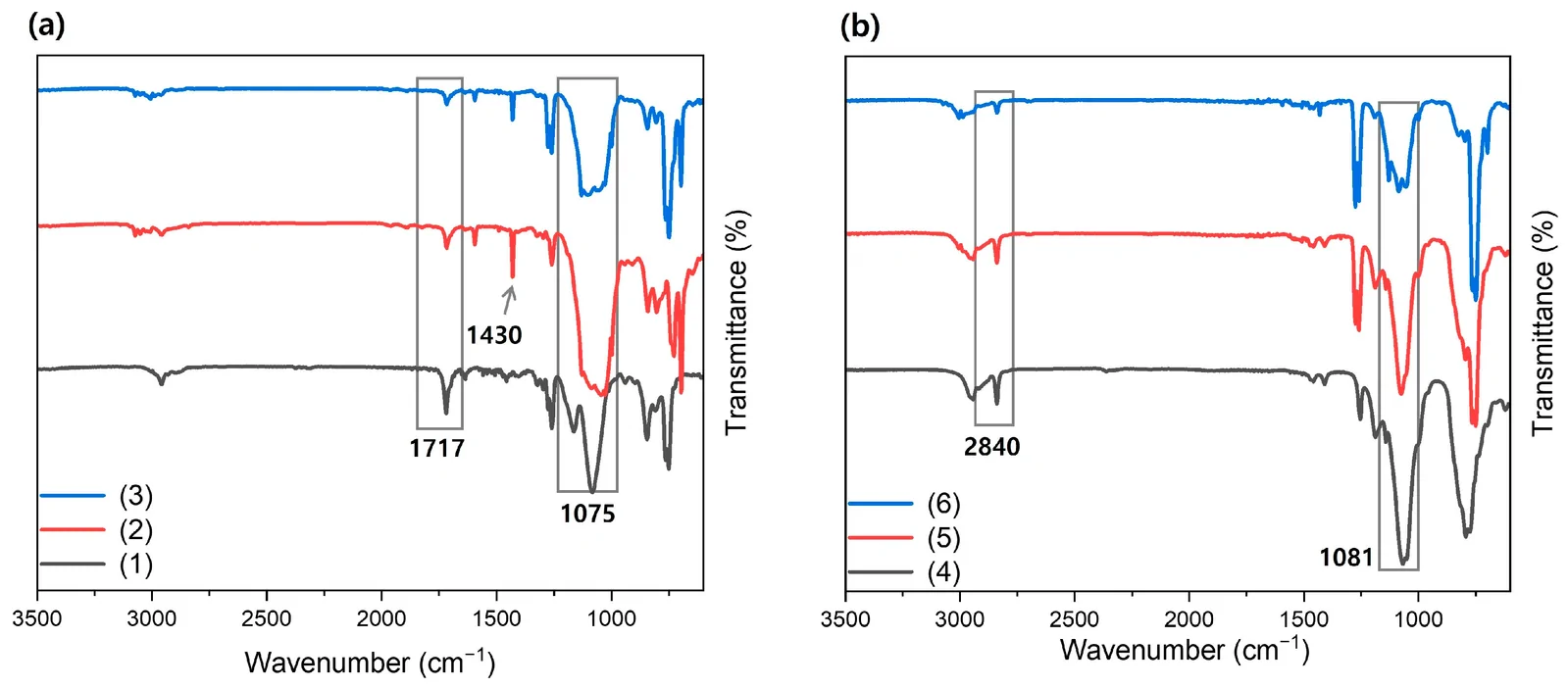
FT-IR spectra of (**a**) methacrylate-functionalized (**1**–**3**) and (**b**) trimethoxysilane-functionalized (**4**–**6**) POSS and DDSQ derivatives.

The FT-IR spectra of compounds **1**–**6** are shown in Figure 3. All synthesized compounds exhibited broad absorption bands in the range of 1017–1110 cm^−1^, which were assigned to the stretching vibrations of Si–O bonds. The presence of these characteristic bands indicates that the siloxane frameworks of POSS and DDSQ were retained during the functionalization reactions. In the spectra of the methacrylate derivatives **1**–**3** (Figure 3a), a strong absorption band was observed at approximately 1717 cm^−1^, corresponding to the C=O stretching vibration of the methacrylate group. This characteristic absorption confirms the introduction of methacrylate functionalities into the POSS and DDSQ frameworks. In contrast, the trimethoxysilane derivatives **4**–**6** exhibited a characteristic strong absorption at approximately 2840 cm^−1^ (Figure 3b), which was associated with the introduced trimethoxysilane groups. The DDSQ derivatives also exhibited weak bands at approximately 1600 and 1430 cm^−1^, which are associated with aromatic C=C vibrations of the phenyl substituents. Because these phenyl groups are retained from the corresponding Si–H-functionalized DDSQ precursors, these bands were used only as supporting evidence for preservation of the DDSQ framework. The introduction of the new peripheral functionalities was instead primarily identified from the characteristic C=O band of **1**–**3** and the methoxy-related absorption of **4**–**6**.

**Figure 4:**
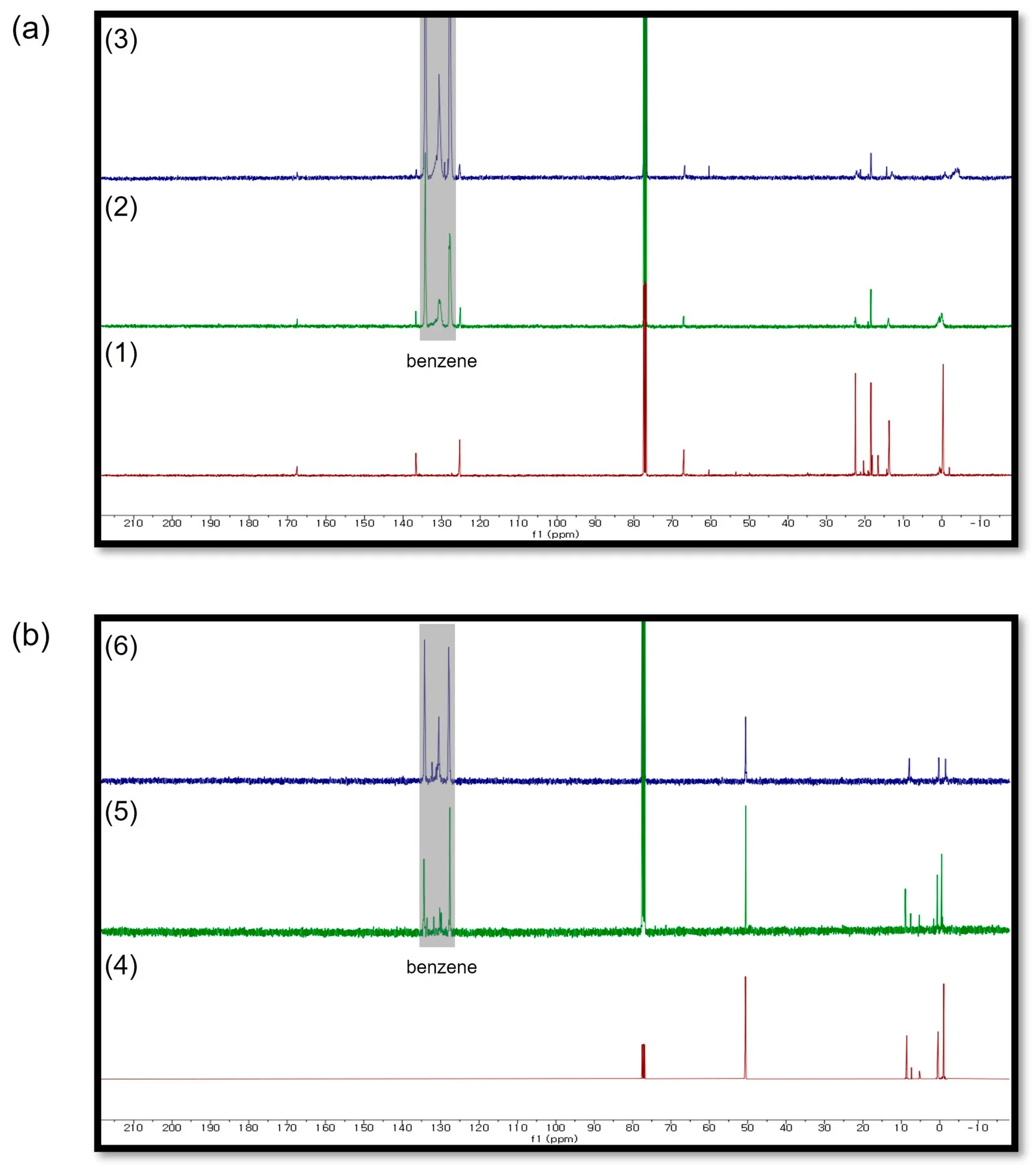
^13^C-NMR spectra of (**a**) methacrylate-functionalized (**1**–**3**) and (**b**) trimethoxysilane-functionalized (**4**–**6**) POSS and DDSQ derivatives.

The structures of the synthesized compounds were further characterized by ^13^C-NMR spectroscopy (Figure 4). The ^13^C-NMR spectra of the methacrylate derivatives showed characteristic resonances at 167.5, 136.6, 125.3, 67.1, 22.4, 18.4, 13.7, and −0.3 ppm (Figure 4a). These signals are associated with the carbon environments of the introduced methacrylate-containing groups and support the successful functionalization of the POSS and DDSQ frameworks. For the trimethoxysilane derivatives, characteristic carbon resonances were observed at 50.6, 8.6, 0.4, and −1.1 ppm (Figure 4b), consistent with the carbon environments of the introduced trimethoxysilane-containing groups. In addition, the DDSQ derivatives exhibited aromatic carbon signals at 134.3, 131.8, 130.2, and 127.6 ppm. These resonances originate from the phenyl groups attached to the DDSQ framework and distinguish the DDSQ derivatives from the corresponding POSS compounds.

**Figure 5:**
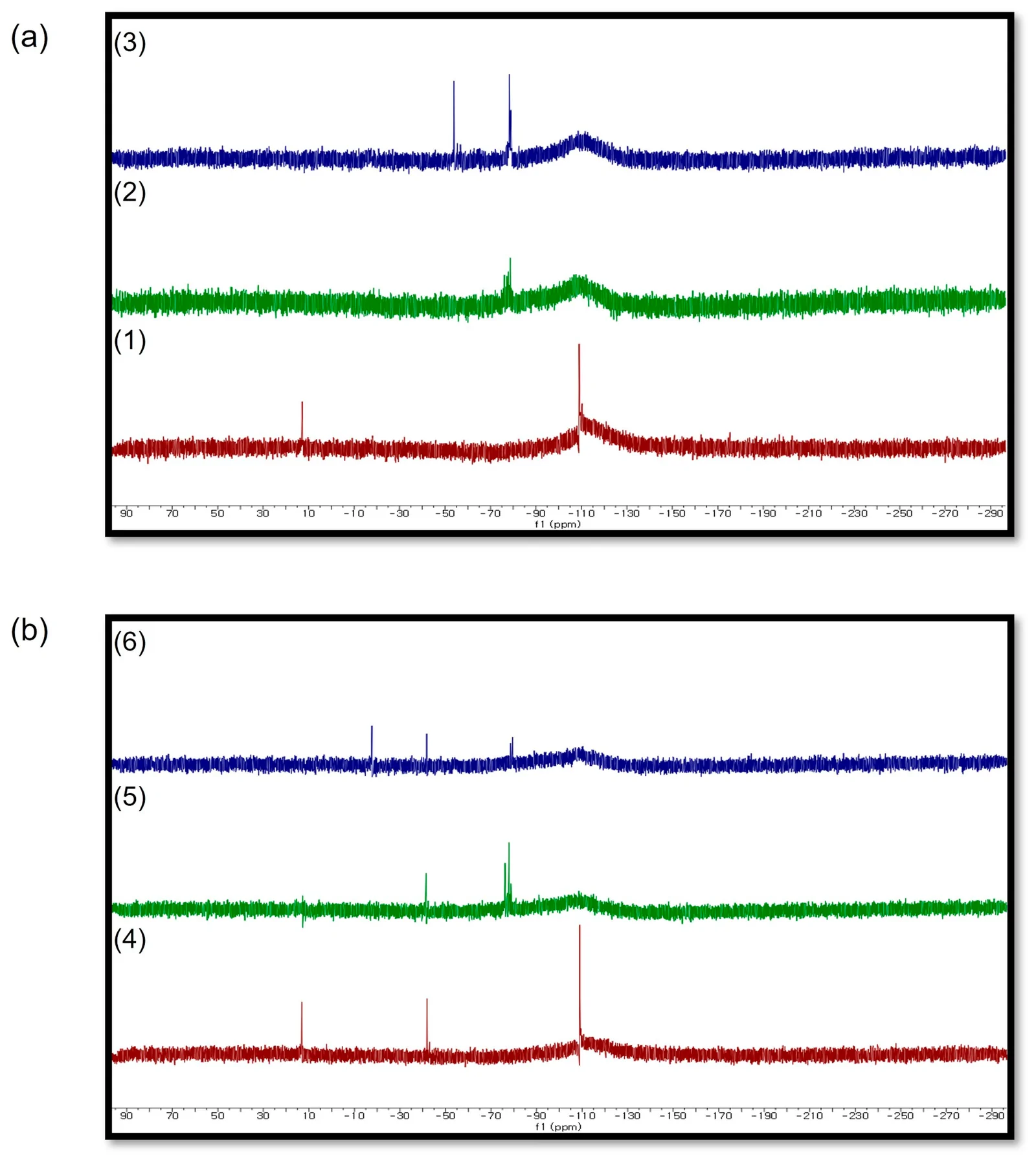
^29^Si-NMR spectra of (**a**) methacrylate-functionalized (**1**–**3**) and (**b**) trimethoxysilane-functionalized (**4**–**6**) POSS and DDSQ derivatives.

The ^29^Si NMR spectra provided further evidence for the preservation of the silsesquioxane frameworks and the successful introduction of the peripheral functional groups (Figure 5). POSS-8-O-Si-Methacryl (**1**) exhibited resonances at 12.9 and −109.0 ppm, assigned to the peripheral R–SiMe_2_–O units and the silicon atoms of the T_8_ cage, respectively. POSS-8-O-Si-Trimethoxysilane (**4**) showed corresponding resonances at 13.2 and −108.9 ppm, together with a signal at −41.7 ppm assigned to the terminal Si(OMe)_3_ groups. The DDSQ derivatives displayed framework-silicon resonances between approximately −76.1 and −79.5 ppm. Within the methacrylate series, the −77.8 ppm resonance reported for DDSQ-4-O-Si-Methacryl (**2**) was associated with its DDSQ-4-O-Si environment, whereas the signal at −54.0 ppm for DDSQ-2-O-Si-Methacryl (**3**) was assigned to the R–Si–O_2_ environment. In the trimethoxysilane series, DDSQ-4-O-Si-Trimethoxysilane (**5**) and DDSQ-2-O-Si-Trimethoxysilane (**6**) exhibited characteristic peripheral-silicon resonances at 12.7 and −17.7 ppm, respectively, and the terminal Si(OMe)_3_ groups of both compounds gave a resonance near −41.7 ppm. Notably, the peripheral silicon resonance shifts from 12.7 ppm for 5 to −17.7 ppm for 6, corresponding to an upfield shift of approximately 30.4 ppm. This difference reflects the change from an R–SiMe_2_–O environment to an R–SiMe–O_2_ environment, in which the additional siloxy oxygen increases the shielding of the silicon center. These complete assignments support both preservation of the POSS and DDSQ frameworks and successful functionalization.

The MALDI-TOF mass spectra of compounds 1–6 are presented in Figures S19–S24, and the calculated and observed *m*/*z* values are summarized in Table 1. For the trimethoxysilane derivatives 4–6, the peaks at *m*/*z* 2223.9445, 1917.0198, and 1472.9825, respectively, were assigned to sodium-adduct molecular ions ([M+Na]^+^), in comparison with calculated values of 2223.4017, 1915.3961, and 1471.1746. Sodium acetate was used as the cationization agent; therefore, these ions were assigned as [M+Na]^+^ species. Because compounds 4–6 contain multiple silicon atoms and exhibit relatively broad isotopic distributions, the most intense MALDI peak may not coincide exactly with the calculated monoisotopic [M+Na]^+^ value. Accordingly, these data were used as complementary molecular-mass evidence rather than as high-resolution exact-mass measurements. Intact sodium-adduct molecular ions were not detected for the methacrylate derivatives **1**–**3**. Instead, their principal peaks at *m*/*z* 1175.3603, 1203.1302, and 1207.1629 were consistent with calculated fragment ions at *m*/*z* 1175.0815, 1203.1702, and 1207.1440, respectively. The absence of intact molecular-ion peaks for 1–3 may be associated with fragmentation during the laser-induced desorption/ionization process, in which the peripheral methacrylate-containing siloxy groups are more susceptible to cleavage. In contrast, the trimethoxysilane derivatives **4**–**6** contain simpler Si–CH_2_CH_2_–Si(OMe)_3_ peripheral groups, which may favor retention of the intact sodium-adduct molecular ions under the same MALDI conditions. However, detailed gas-phase fragmentation pathways were not investigated in the present study. Therefore, the MALDI-TOF fragment ions observed for **1**–**3** were regarded as supportive, rather than definitive, molecular-mass evidence and were interpreted together with the ^1^H-/^13^C-NMR, ^29^Si-NMR, and FT-IR data. Accordingly, the MALDI-TOF results provide complementary support for the proposed structures, while the structural assignments are based on the combined spectroscopic evidence.

Overall, the FT-IR, ^13^C-NMR, and ^29^Si-NMR results consistently support the successful synthesis of the methacrylate- and trimethoxysilane-functionalized POSS and DDSQ derivatives. In particular, the spectroscopic data confirm both the retention of the characteristic silsesquioxane frameworks and the successful introduction of the desired organic functional groups. The distinct ^29^Si-NMR features of the DDSQ-4-O-Si and DDSQ-2-O-Si derivatives further demonstrate that the different DDSQ architectures were maintained during the functionalization reactions.

## 4. Conclusions

In summary, a systematic series of six functional silsesquioxane derivatives based on three molecular architectures, POSS-8, DDSQ-4, and DDSQ-2, was prepared through hydrosilylation of the corresponding Si–H-functionalized precursors. The resulting series comprises three methacrylate-functionalized derivatives and three trimethoxysilane-functionalized derivatives spanning different framework architectures and peripheral functional-group densities. The products were obtained in good yields and characterized by FT-IR, ^1^H and ^13^C NMR, ^29^Si NMR, and MALDI-TOF mass spectrometry. The combined spectroscopic data support successful functionalization while retaining the characteristic POSS and DDSQ frameworks.

The principal contribution of this study is the establishment of a well-defined molecular precursor library in which silsesquioxane framework architecture and peripheral functionality are systematically organized within a common synthetic framework. Compounds **1**–**6** should therefore be regarded as potential molecular precursors for subsequent studies rather than as materials with demonstrated polymerization or sol–gel performance. The present data do not establish quantitative architecture-dependent reaction kinetics, polymerization behavior, hydrolysis/condensation behavior, or material properties. These aspects will require dedicated studies under standardized reaction and processing conditions.

## Figures and Tables

**Table 1:** Calculated and observed MALDI-TOF MS values for compounds **1**–**6**. Fragment-ion assignments for compounds **1**–**3** are provided as supportive mass-spectrometric evidence.

	Calculated [M+Na]^+^ (*m*/*z*)	Observed (*m*/*z*)	Calculated Fragment Mass (*m*/*z*)
(**1**)	2047.5017	1175.3603	1175.0815
(**2**)	1827.4461	1203.1302	1203.1702
(**3**)	1427.1996	1207.1629	1207.144
(**4**)	2223.4017	2223.9445	-
(**5**)	1915.3961	1917.0198	-
(**6**)	1471.1746	1472.9825	-

## Data Availability

The original contributions presented in this study are included in the article/Supplementary Material. Further inquiries can be directed to the corresponding authors.

## Supplementary Materials

The following supporting information can be downloaded at https://www.mdpi.com/article/10.3390/nano16211343/s1. Figure S1. ^1^H NMR spectrum of POSS-8-O-Si-Methacryl (**1**). Figure S2. ^1^H NMR spectrum of DDSQ-4-O-Si-Methacryl (**2**). Figure S3. ^1^H NMR spectrum of DDSQ-2-O-Si-Methacryl (**3**). Figure S4. ^1^H NMR spectrum of POSS-8-O-Si-Trimethoxysilane (**4**). Figure S5. ^1^H NMR spectrum of DDSQ-4-O-Si-Trimethoxysilane (**5**). Figure S6. ^1^H NMR spectrum of DDSQ-2-O-Si-Trimethoxysilane (**6**). Figure S7. ^13^C NMR spectrum of POSS-8-O-Si-Methacryl (**1**). Figure S8. ^13^C NMR spectrum of DDSQ-4-O-Si-Methacryl (**2**). Figure S9. ^13^C NMR spectrum of DDSQ-2-O-Si-Methacryl (**3**). Figure S10. ^13^C NMR spectrum of POSS-8-O-Si-Trimethoxysilane (**4**). Figure S11. ^13^C NMR spectrum of DDSQ-4-O-Si-Trimethoxysilane (**5**). Figure S12. ^13^C NMR spectrum of DDSQ-2-O-Si-Trimethoxysilane (**6**). Figure S13. ^29^Si NMR spectrum of POSS-8-O-Si-Methacryl (**1**). Figure S14. ^29^Si NMR spectrum of DDSQ-4-O-Si-Methacryl (**2**). Figure S15. ^29^Si NMR spectrum of DDSQ-2-O-Si-Methacryl (**3**). Figure S16. ^29^Si NMR spectrum of POSS-8-O-Si-Trimethoxysilane (**4**). Figure S17. ^29^Si NMR spectrum of DDSQ-4-O-Si-Trimethoxysilane (**5**). Figure S18. ^29^Si NMR spectrum of DDSQ-2-O-Si-Trimethoxysilane (**6**). Figure S19. MALDI-TOF Mass spectrum of POSS-8-O-Si-Methacryl (**1**). Figure S20. MALDI-TOF Mass spectrum of DDSQ-4-O-Si-Methacryl (**2**). Figure S21. MALDI-TOF Mass spectrum of DDSQ-2-O-Si-Methacryl (**3**). Figure S22. MALDI-TOF Mass spectrum of POSS-8-O-Si-Trimethoxysilane (**4**). Figure S23. MALDI-TOF Mass spectrum of DDSQ-4-O-Si-Trimethoxysilane (**5**). Figure S24. MALDI-TOF Mass spectrum of DDSQ-2-O-Si-Trimethoxysilane (**6**). References [37,38,39,40,41,42,43] are cited in the supplementary materials.
