# Supplementary material for: Systematic Design and Synthesis of Methacrylate- and Trimethoxysilane-Functionalized POSS and DDSQ Nanostructures
Source: Nanomaterials (Basel). 2026 Oct 23;16(21):1343. doi: 10.3390/nano16211343 (PMC13765242; doi:10.3390/nano16211343)
Supplement: Supplementary file 1 [file nanomaterials-16-01343-s001.zip › nanomaterials-4599070-supplementary.pdf]

Supporting Information

Supplementary Materials for

Systematic Design and Synthesis of Methacrylate- and Tri-methoxysilane-Functionalized POSS and DDSQ Nanostruc-tures

Bu-Hyun Moon[1] · Eo Jin Oh[2] · Hea Jung Park[2,*] · Dong-Soo Shin[1,*]

[1] R & D Center, Unis Co., Ltd., 1394, Nakdong-daero, Pusan, 46907, S. Korea

[2] Department of Chemistry, Changwon National University, Changwon, 51140, S. Korea

* Email: hjpark@changwon.ac.kr / dsshin@uniss.asia

Table of Contents

I. General experimental details S2

II. Synthesis of POSS-8-O-Si-Methacryl (**1**) and POSS-8-O-Si-Trimethoxysilane (**4**) S2

III. Synthesis of DDSQ-4-O-Si-Methacryl (**2**) and DDSQ-4-O-Si-Trimethoxysilane (**5**) S4

IV. Synthesis of DDSQ-2-O-Si-Methacryl (**3**) and DDSQ-2-O-Si-Trimethoxysilane (**6**) S6

V. Data of ^{1}H and ^{13}C NMR, IR and MALDI-TOF Mass Spectroscopy.....................................S8

VI. References...S21

I. General experimental details

All experiments were performed in round-bottom flasks equipped with a magnetic stirrer. All chemicals were purchased from Tokyo Chemical Industries (TCI) and used as received from Sigma-Aldrich. Solvents were purchased from Daejung and dried according to the reported method. Column chromatography was performed on silica gel (60-120 mesh). Solvent evaporation was performed under reduced pressure using a rotary evaporator. NMR spectra were measured on a BRUKER AVANCE 400 (BRUKER, Germany) spectrometer in CDCl3 using residual low-purity solvent ($CHCl_3$, ^{1}H7.26/^{13}C77.0) and tetramethylsilane (TMS, δ 0) as internal standards, and values are expressed in parts per million (ppm). IR spectra were recorded on an FT-IR-6300 (JASCO, Japan) spectrometer.

Intermediates 7, 8, 9, 10, and 11 were synthesized according to previously reported procedures, and their ^{1}H-NMR spectra were consistent with the reported data. [1-5] Compounds 1, 3, and 4 were also prepared following literature methods, and their ^{1}H-NMR spectra were in good agreement with the previously reported values. [6-9]

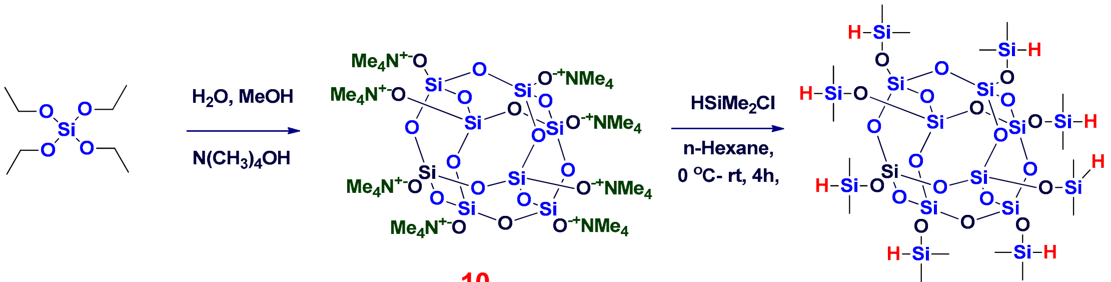

10

7

II. Synthesis of POSS-8-O-Si-Methacryl (1) and POSS-8-O-Si-Trimethoxysilane (4)

(a) Synthesis of octakis(tetramethylammonium)-T_8-silsesquioxane (10)

At a reaction temperature of 0 °C, a 25 wt% methanolic solution of tetramethylammonium hydroxide (27.7 mL, 0.26 mol), MeOH (7.0 mL, 0.33 mol), and deionized water (10 mL, 9.56 mol) are sequentially added to a two-neck flask (250 mL), and a solution of tetraethoxysilane (tetraethyl orthosilicate (14.7 mL, 0.065 mol, 13.68 g, d=0.933) dissolved in MeOH (10 mL) is slowly added. Raise the temperature of the reaction solution to RT, stir for 24 hours, and check the progress of the reaction using TLC. After confirming the reaction, proceed with the next reaction.

(b) Synthesis of octa(hydridodimethylsiloxy)octasil sesquioxane, POSS-8-O-Si-H (7)

At a reaction temperature of 0 °C, in a two-neck flak (2 L), add n-Hexane (700 mL), then add

chlorodimethylsilane (75.0 g, 0.79 mol). To the reaction solution, the previously synthesized octakis(tetramethylammonium)-T_8-silsesquioxane (**10**, 74 g, 0.065 mol, 1 equiv.) solution is slowly added dropwise using a dropping funnel. Slowly raise the temperature of the reaction solution to RT, stir the reaction solution for 12 hours, and then check the progress of the reaction using TLC (hexane layer and water layer). After the reaction was completed, the organic layer was separated and distilled under reduced pressure. The obtained solid compound was washed three times with MeOH to obtain POSS-8-O-Si-H (**7**, 61.2 g, 91.8% yield). ^{1}H NMR (400 MHz, $CDCl_3$): δ 4.73 (hept, *J* = 2.8 Hz, 8H), 0.25 (d, *J* = 2.8 Hz, 48H).; IR (Neat): λ_{max} 2964, 2140, 1254, 1069, 889, 767, 542 cm^{-1}.

(c) Synthesis of POSS-8-O-Si-Methacryl (1)

In a two-neck flask (250 mL), POSS-8-O-Si-H (**7**) (3.0 g, 2.95 mmol) was placed at room temperature, and toluene (30 mL) was added. A solution of ally methacrylate (3.72 g, 29.49 mmol, 10 equiv.) dissolved in toluene (10 mL) was added to the reaction solution, and Karstedt's catalyst (0.07 mL) (Platinum(0)-1,3-divinyl-1,1,3,3-tetramethyldisiloxane complex solution) was added to the reaction solution. The reaction temperature was raised to 60 ℃ (internal temperature), and the progress of the reaction was checked using TLC from 3 hours of reaction time. After completion of the reaction, the solvent was removed using a rotary evaporator under reduced pressure, and the product was separated using column chromatography to obtain POSS-8-O-Si-Methacryl (**1**) (4.83 g, yield 81%). ^{1}H NMR (400 MHz, $CDCl_3$): δ 0.2 (s, Si-CH_3, 48H), 0.6(m, Si-CH_2-, 16H), 1.4-1.7 (m, -CH_2-CH_2-, 16H), 1.9 (s, =C-CH_3, 24H), 4.1 (m, -CH_2-O-, 16H), 5.6 (d, -CH_2, 8H), 6.0 (d, -CH_2, 8H): ^{13}C NMR (100 MHz, $CDCl_3$): δ 167.54, 136.65, 125.26, 67.09, 22.44, 18.42, 13.74, -0.32; ^{29}Si NMR (79.5 MHz, $CDCl_3$) δ 12.9, −109.0; FTIR (neat): ν_{max} 3003 (sp^2 C-H str, w), 2961 (sp^3 C-H str, w), 1721 (C=O str, m), 1260 (Si-CH_3 str, m), 1084 (O-Si-O str, s), 750 (Si-C str, s) cm^{-1}.

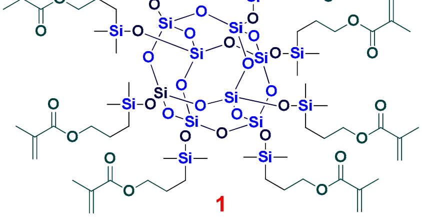

1

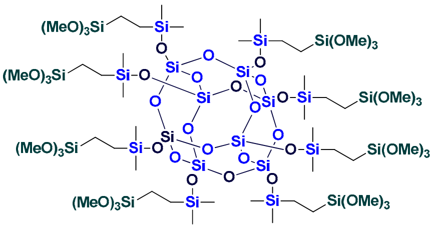

4

(d) Synthesis of POSS-8-O-Si-Trimethoxysilane (**4**)

POSS-8-O-Si-H (**7**) (1.0 g, 0.98 mmol, 1 equiv.) two-neck flask (100 mL) at room temperature,

and toluene (20 mL) was added. Trimethoxyvinylsilane (0.73 g, 4.9 mmol, 5 equiv.) was added to the reaction solution, and Karstedt's catalyst (three drops) was added to the reaction solution. The reaction temperature was raised to 70 ℃ (internal temperature), and the progress of the reaction was checked using TLC from 3 hours of reaction time. After completion of the reaction, the solvent was removed using a rotary evaporator under reduced pressure, and the product was separated using column chromatography to obtain POSS-8-O-Si-trimethoxysilane (**4**) (1.84 g, yield 85%). ^{1}H NMR (400 MHz, $CDCl_3$): δ 0.15 (s, $Si(CH_3)_2$ 48H), 0.5–0.70 (m, $SiCH_2$, 16H), 1.2–1.4 (m, $CH_2Si(OCH_3)_3$, 16H), 3.5–3.6 (s, $Si(OCH_3)_3$, 72H); ^{13}C NMR (100 MHz, $CDCl_3$): δ 50.61, 8.58, 0.42, -1.08; ^{29}Si NMR (79.5 MHz, $CDCl_3$) δ 13.2, −41.7, −108.9; FTIR (neat): ν_{max} 2945 (sp^3 C-H str, w), 2839 (Si-OMe str, w), 1254 ($Si-CH_3$ str, w), 1070 (O-Si-O str, s), 795 (Si-C str, s) cm^{-1}.

III. Synthesis of DDSQ-4-O-Si-Methacryl (2) and DDSQ-4-O-Si-Trimethoxysilane (5)

(a) Synthesis of DDSQ-4-ONa (**11**)

At room temperature, trimethoxy(phenyl)silane (50.0 g, 252.16 mmol, 1 equiv.) was added first to a one-neck flask (500 mL), then isopropyl alcohol (250 mL, bp 82.3 ℃), deionized water (5.67 g, 0.315 mol, 1.5 equiv.), and NaOH (6.66 g, 167 mmol, 0.66 equiv.) were added in sequence, and then refluxed. The reaction temperature was raised to 165℃, and after 5 hours of reaction, the progress of the reaction was checked using TLC. The reaction temperature was lowered to RT, stirred at RT for 15 hours, and the solid compound obtained after completion of the reaction was filtered. The solid compound obtained was washed with IPA and dried with a vacuum pump (using a heat gun) to obtain a white solid product, DSQ-4-ONa (**11**) (32 g, yield 90%).

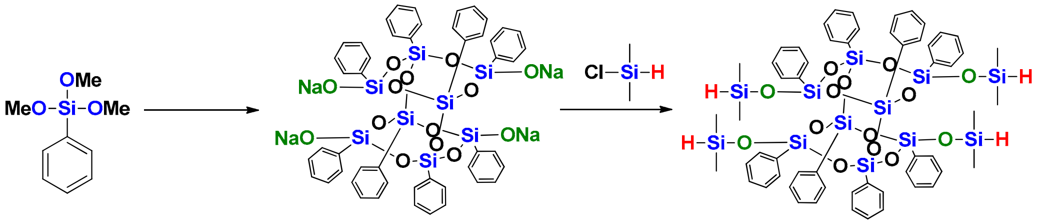

11

8

(b) Synthesis of DDSQ-4-O-Si-H (**8**)

At room temperature, in a two-neck flask (500 mL), DDSQ-4-ONa (**11**) (14.6 g, 12.61 mmol, 1 equiv.) was added THF (130 mL), triethylamine (6.4 g, 63.05 mmol, 5 equiv.) and stirred. A solution of chlorodimethylsilane (6.0 g, 63.05 mmol, 5 equiv.) dissolved in THF (20 mL) was slowly added to the reaction solution while stirring. The reaction progress of the reaction solution

was confirmed by TLC, and after the reaction was completed, the solvent (THF) was removed through a reduced pressure rotary evaporator to work up, and the obtained compound was recrystallized to obtain DDSQ-4-O-Si-H (**8**) (15.3 g, yield 93%). ^{1}H NMR (400 MHz, $CDCl_3$): δ 0 .1 ppm (d, $Si(CH_3)_2$, 24H), 4.7 (m, Si-H, 4H), 7.2 (m, phenyl. 40H) ppm.

(c) Synthesis of DDSQ-4-O-Si-Methacryl (**2**)

At room temperature, DDSQ-4-O-Si-H (**8**) (3.0 g, 2.30 mmol, 1 equiv.) was added to a two-neck flask (250 mL), toluene (30 mL) was added, and stirred. A solution of allylic methacrylate (1.5 g, 11.5 mmol, 5 equiv.) dissolved in toluene (10 mL) was added to the reaction solution, and Karstedt's catalyst (0.05 mL) was added to the reaction solution. The reaction temperature was raised to 60 ℃ (internal temperature), and the progress of the reaction was checked using TLC from 3 hours of reaction time. After completion of the reaction, the solvent was removed using a rotary evaporator under reduced pressure, and the product was separated by a column chromatography to obtain DDSQ-4-O-Si-Methacryl (**2**) (3.5 g, yield 85%). ^{1}H NMR (400 MHz, $CDCl_3$): δ 0.1-0.3 (s, $Si(CH_3)_3$, 24H), 0.4-0.6 (m, Si-CH_2-, 8H), 1.4-1.7 (m, -CH_2-, 8H), 1.8-2.0 (s, -$C(CH_3)$=, 12H), 4.0-4.3 (t, O-CH_2-, 8H), 5.5-6.1 (br. s, =CH_2, 8H), 7.1-7.8 (m, Ar-H, 40H); ^{13}C NMR (100 MHz, $CDCl_3$): δ 167.5, 136.7, 134.2, 130.6, 130.5, 127.9, 125.2, 67.1, 22.4, 18.4, 13.9, 0.56; ^{29}Si NMR (79.5 MHz, $CDCl_3$) δ −76.1, −77.8, −78.7; FTIR (neat): ν_{max} 3100-3000 (sp^2 C-H str, w), 2957 (sp^3 C-H str, w), 1717 (C=O str, w), 1261 (Si-CH_3, str, w), 1047 (O-Si-O str, s), 696 (Si-C str, s) cm^{-1}.

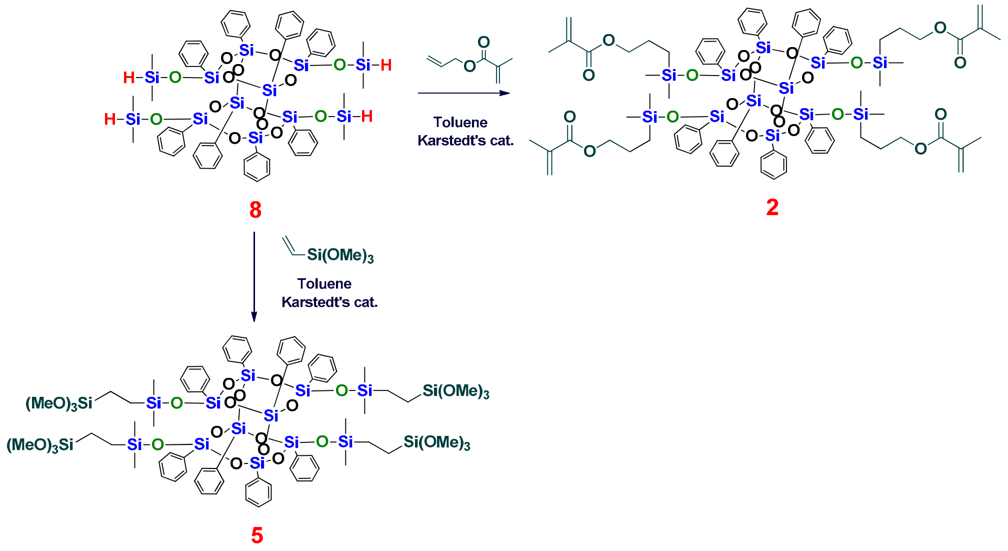

(d) Synthesis of DDSQ-4-O-Si-Trimethoxysilane (**5**)

At RT, DDSQ-4-O-Si-H (**8**) (0.98 g, 0.75 mmol, 1 equiv.) was added to a two-neck flask, toluene (20 mL) was added, and stirred. Trimethoxyvinylsilane (0.56 g, 3.78 mmol, 5 equiv., MW 148.23)

was added to the reaction solution, and Karstedt's catalyst (three drops) was added. The reaction temperature was raised to 70 ℃ (internal temperature), and the progress of the reaction was checked using TLC after 3 hours of reaction time. After completion of the reaction, the solvent was removed using a rotary evaporator under reduced pressure, and the product was separated by a column chromatography to obtain DDSQ-4-O-Si-Trimethoxysilane (**5**) (1.2 g, yield 86%). ^{1}H NMR (400 MHz, $CDCl_3$): δ 0.1 (s, $Si(CH_3)_2$, 24H), 0.6 (m, $SiCH_2$, 8H), 1.4 (m, $CH_2Si(OMe)_3$, 3.4 (s, $Si(OCH_3)_3$, 36H), 7.1-7.6 (m, phenyl, 40H); ^{13}C NMR (100 MHz, $CDCl_3$): δ 134.4, 131.8, 130.3, 127.6, 50.5, 8.9, 0.6, -0.5; ^{29}Si NMR (79.5 MHz, $CDCl_3$) δ 12.7, −41.5, −76.2, −77.9; FTIR (neat): ν_{max} 3005 (sp^2 C-H str, w), 2941 (sp^3 C-H str, w), 2841 (Si-OMe str, w), 1260 (Si-CH_3 str, m), 1076 (O-Si-O str, s), 750 (Si-C str, s) cm^{-1}.

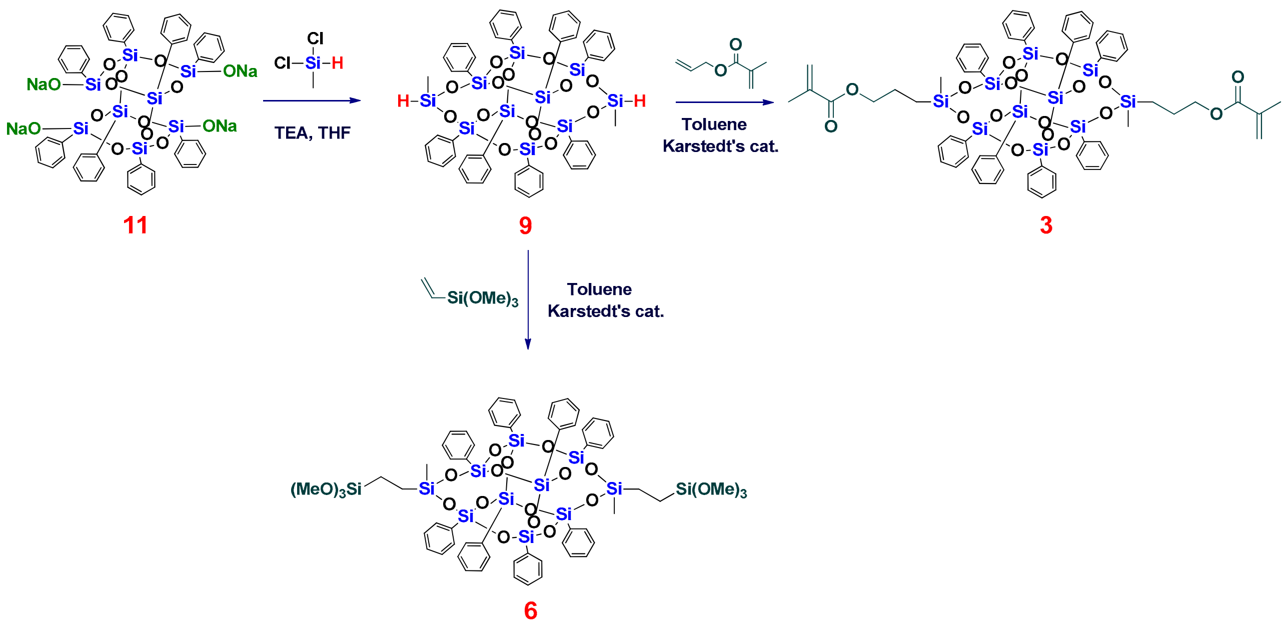

IV. Synthesis of DDSQ-2-O-Si-Methacryl (3) and DDSQ-2-O-Si-Trimethoxysilane (6)

(a) Synthesis of DDSQ-2-O-Si-H (**9**)

At room temperature, in a two-neck flask (500 mL), DDSQ-4-ONa (**11**) (15 g, 12.96 mmol) was added THF (130 mL), triethylamine (6.6 g, 64.8 mmol, 5 equiv.) was added, and the mixture was stirred. A solution of dichloro(methyl)silane (4.5 g, 38.9 mmol, 3 equiv.) dissolved in THF (20 mL) was slowly added to the reaction solution while stirring. The reaction progress of the reaction solution was checked by temperature TLC, and after the reaction was completed, the solvent (THF) was removed through a reduced pressure rotary evaporator to work up, and the obtained compound was recrystallized to obtain DDSQ-2-O-Si-H (**9**) (13.7g, yield 92%). ^{1}H NMR (400 MHz, $CDCl_3$): δ 0.2-0.4 (d, $SiCH_3$, 6H), 4.7-5.1 (m, SiH, 2H), 7.1-7.7 (m, phenyl, 40H).

(b) Synthesis of DDSQ-2-O-Si-Methacryl (**3**)

At RT, DDSQ-2-O-Si-H (**9**) (2.7 g, 2.30 mmol, 1 equiv.) was added to a two-neck flask (250 mL), and toluene (30 mL) was added. A solution of allylic methacrylate (1.64 g, 13 mmol, 5 equiv.) dissolved in toluene (10 mL) was added to the reaction solution, and Karstedt's catalyst (0.05 mL) was added to the reaction solution. The reaction temperature was raised to 60 ℃ (internal temperature) and stirred. The progress of the reaction was checked using TLC from 3 hours of reaction time, and after completion of the reaction, the solvent was removed using a rotary evaporator under reduced pressure, and the product was separated using a column chromatography to obtain DDSQ-2-O-Si-Methacryl (**3**) (3.2 g, yield 87%). ^{1}H NMR (400 MHz, $CDCl_3$): δ 0.1-0.3 (s, $SiCH_3$, 6H), 0.4-0.7 (m, $SiCH_2$, 4H), 1.4-1.7 (m, C-CH_2-C, 4H), 1.8=2.0 (s, $C(CH_3)=CH_2$, 6H), 3.9-4.2 (t, CH_2OCO, 4H), 5.5-5.8 (br s, $=CH_2$, 2H), 6.0-6.2 (br s, $=CH_2$, 2H), 7.1-7.7 (m, phenyl, 40H); ^{13}C NMR (100 MHz, $CDCl_3$): δ 167.5, 136.6, 134.2, 130.8, 130.7, 127.8, 125.26, 66.8, 22.2, 18.4, 14.3, -0.8; ^{29}Si NMR (79.5 MHz, $CDCl_3$) δ −54.0, −78.3, −78.9; FTIR (neat): ν_{max} 3100-3020 (sp^2 C-H str, w), 3000 (sp^3 C-H str, w), 1717 (C=O str, w), 1260 (Si-CH_3 str, m), 1092 (O-Si-O str, s), 750 (Si-C str, s) cm^{-1}.

(c) Synthesis of DDSQ-2-O-Si-trimethoxysilane (**6**)

At RT, DDSQ-2-O-Si-H (**9**) (1.0 g, 0.9 mmol, 1 equiv.) was added to a two-neck flask, and toluene (20 mL) was added. Trimethoxyvinylsilane (0.4 g, 2.7 mmol, 3 equiv.) was added to the reaction solution, followed by the addition of Karstedt's catalyst (three drops), and the reaction temperature was raised to 70 ℃ (internal temperature) and stirred. After 3 hours of reaction, the progress of the reaction was checked by TLC, and after completion of the reaction, work-up was performed, the solvent was removed by a rotary evaporator under reduced pressure, and the product was separated by a column chromatography to obtain DDSQ-2-O-Si-trimethoxysilane (**6**) (1.2 g, yield 91%). ^{1}H NMR (400 MHz, $CDCl_3$): δ 0.1-0.3 (s, $SiCH_3$, 6H), 0.4-0.7 (m, $SiCH_2$, 4H), 1.1-1.5 (m, $CH_2Si(OMe)_3$, 4H), 3.4-3.6 (s, $Si(OCH_3)_3$, 18H), 7.1-7.7 (m, phenyl, 40H); ^{13}C NMR (100 MHz, $CDCl_3$): δ 134.2, 132.2, 130.5, 127.9, 50.6, 7.9, 0.3, -1.6; ^{29}Si NMR (79.5 MHz, $CDCl_3$) δ −17.7, −41.7, −78.7, −79.5; FTIR (neat): ν_{max} 3007 (sp^2 C-H str, w), 2988 (sp^3 C-H str, w), 2837 (Si-OMe str, w), 1275 (Si-CH_3 str, m), 1086 (O-Si-O str, m), 750 (Si-C str, s) cm^{-1}.

V. Data of ^{1}H and ^{13}C-NMR, IR and MALDI-TOF Mass Spectroscopy

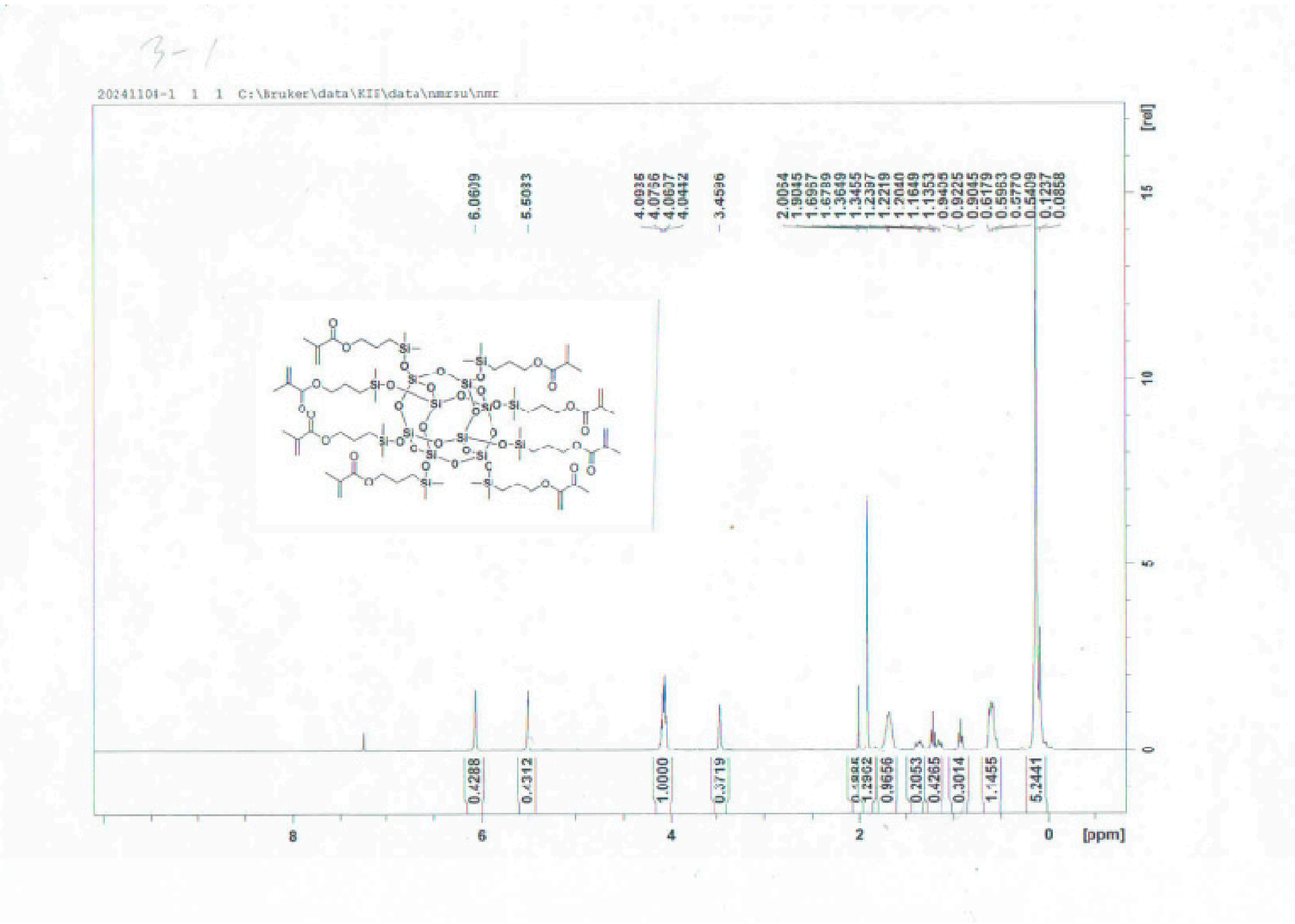

Figure S1. ^{1}H NMR spectrum of POSS-8-O-Si-Methacryl (1).

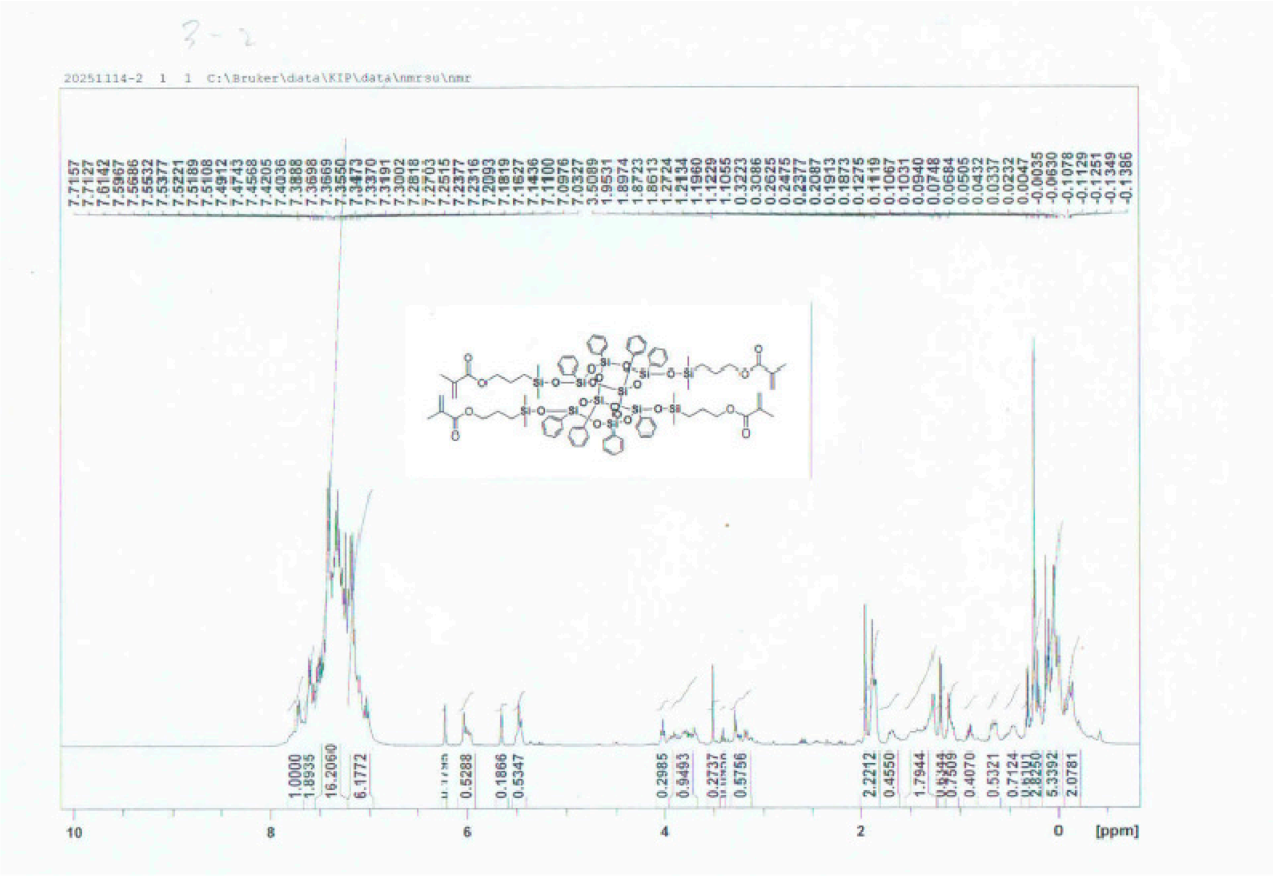

Figure S2. ^{1}H NMR spectrum of DDSQ-4-O-Si-Methacryl (2)

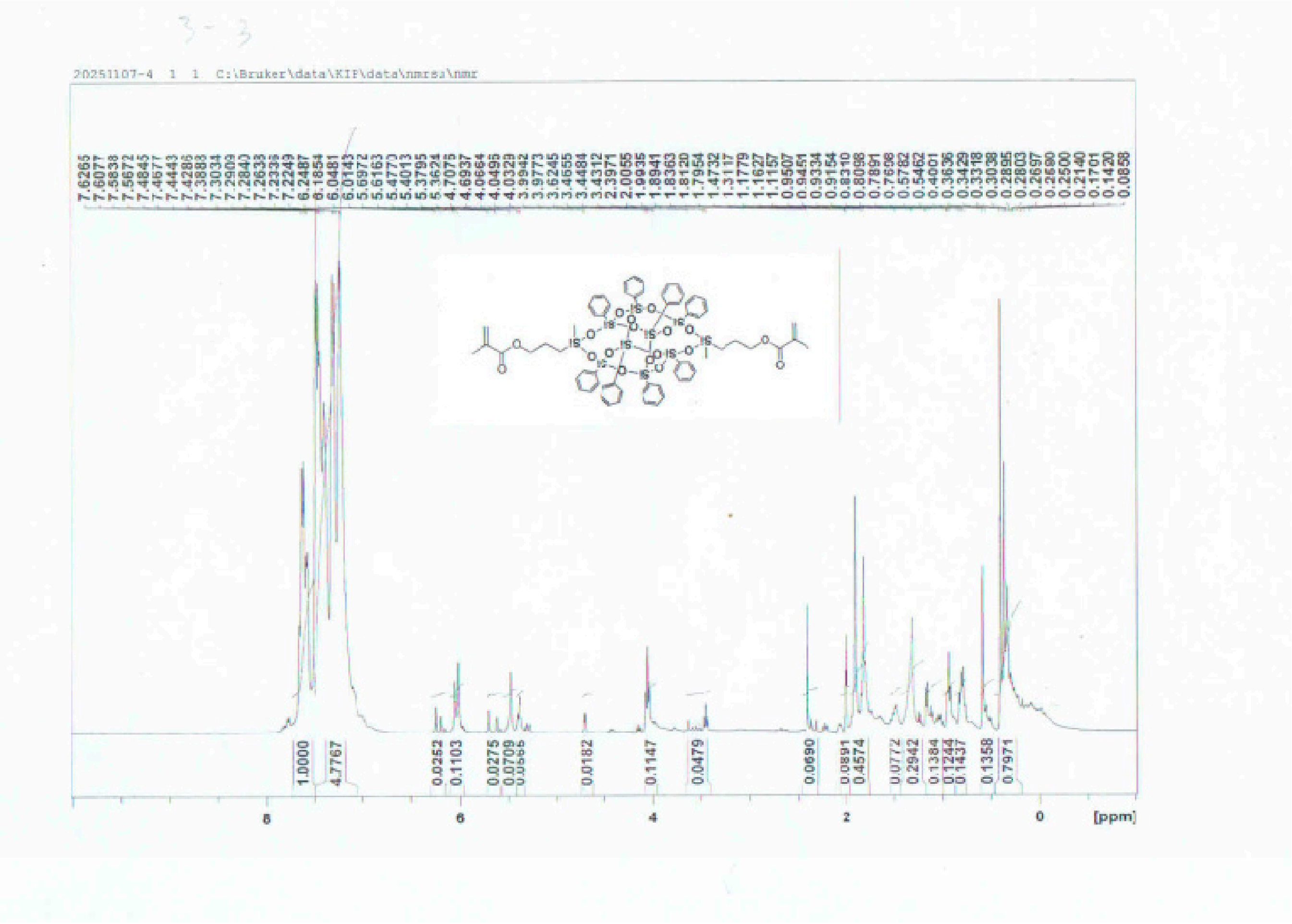

Figure S3. ^{1}H NMR spectrum of DDSQ-2-O-Si-Methacryl (3).

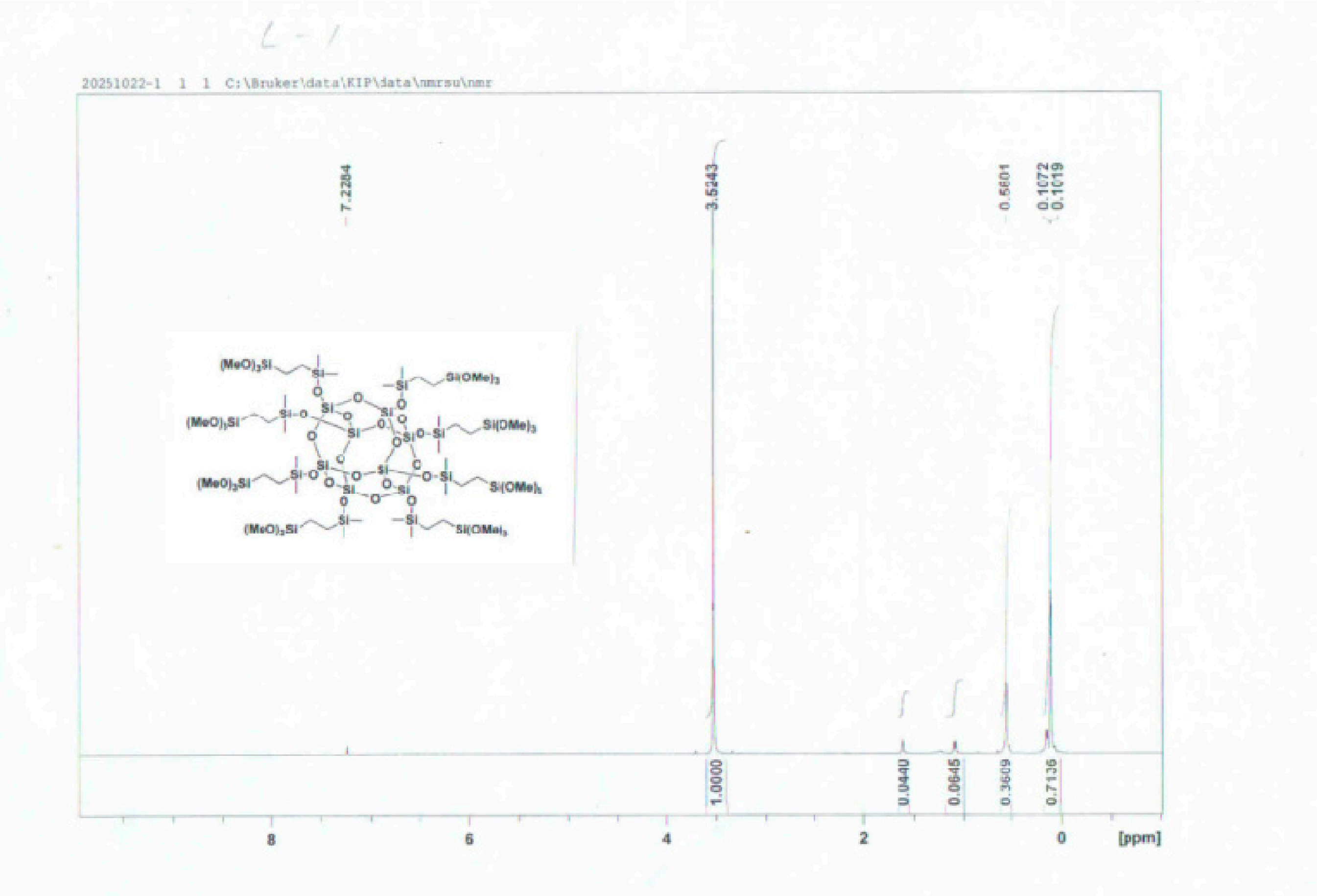

Figure S4. ^{1}H NMR spectrum of POSS-8-O-Si-Trimethoxysilane (4).

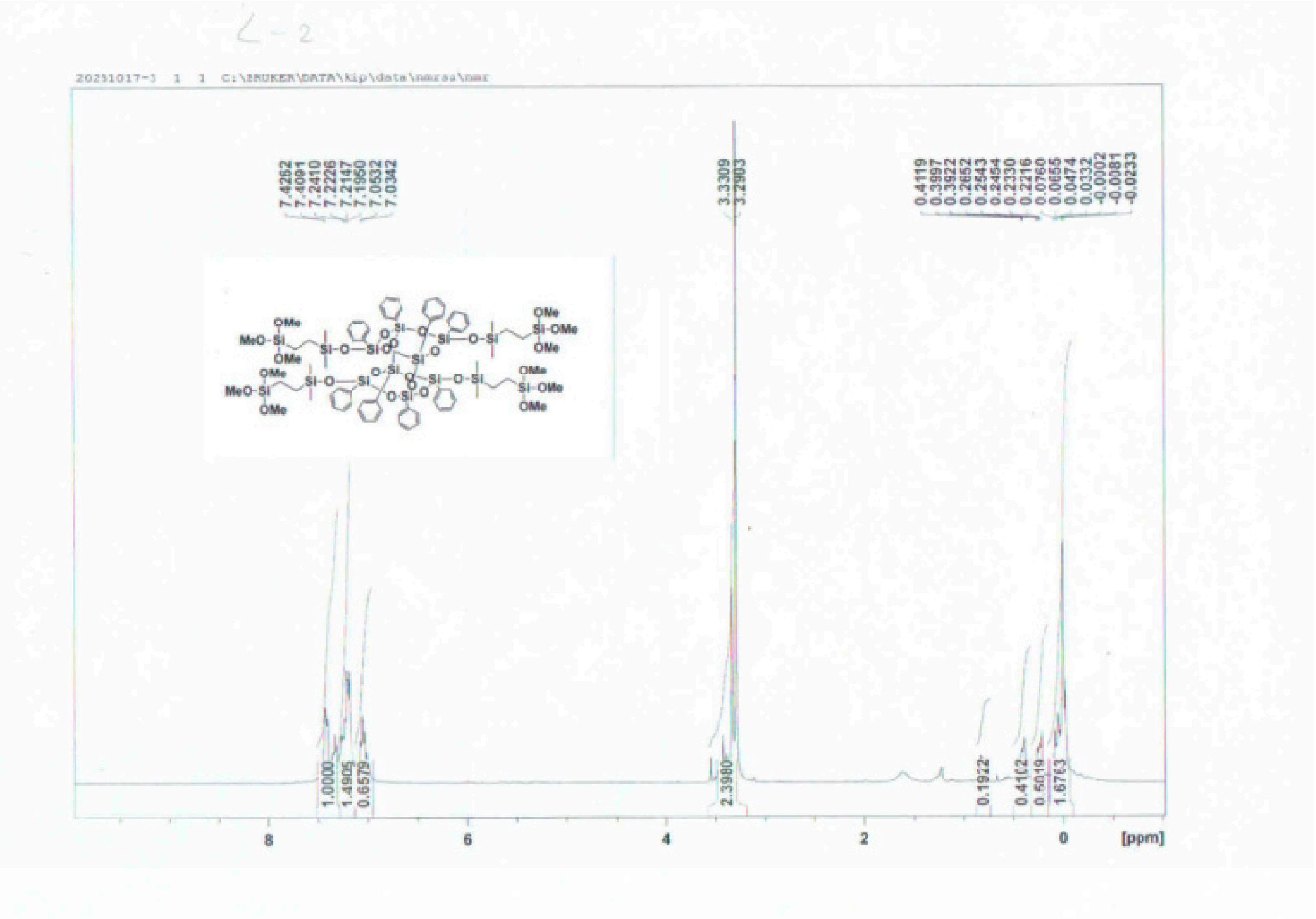

Figure S5. ^{1}H NMR spectrum of DDSQ-4-O-Si-Trimethoxysilane (5).

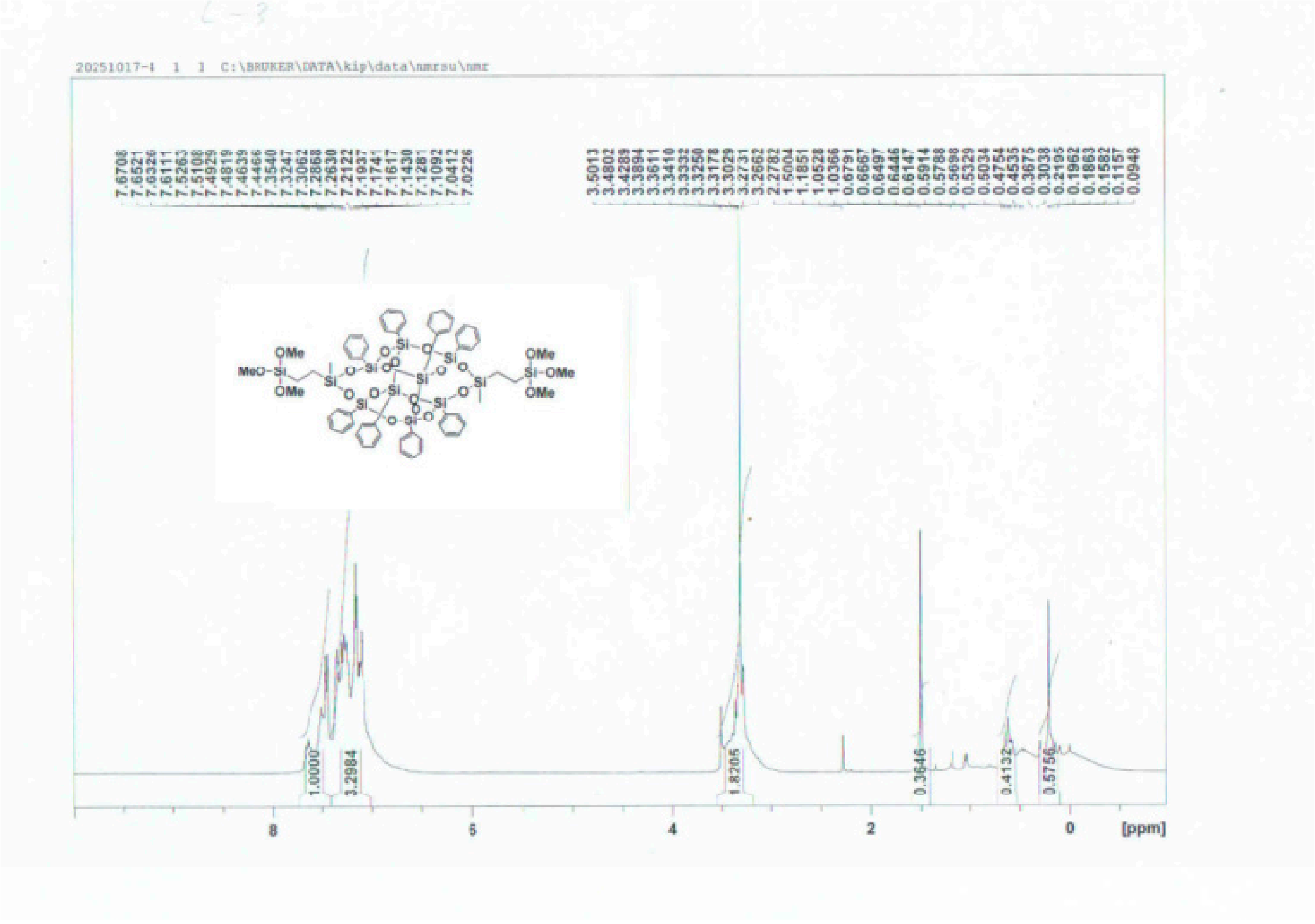

Figure S6. ^{1}H NMR spectrum of DDSQ-2-O-Si-Trimethoxysilane (6).

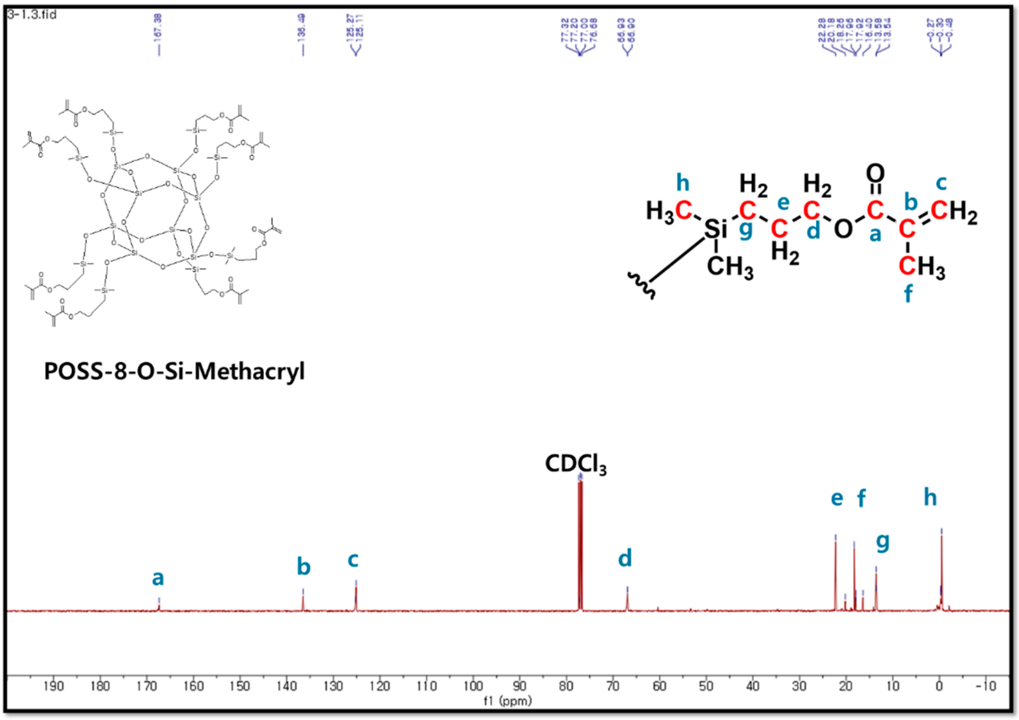

Figure S7. ^{13}C NMR spectrum of POSS-8-O-Si-Methacryl (1).

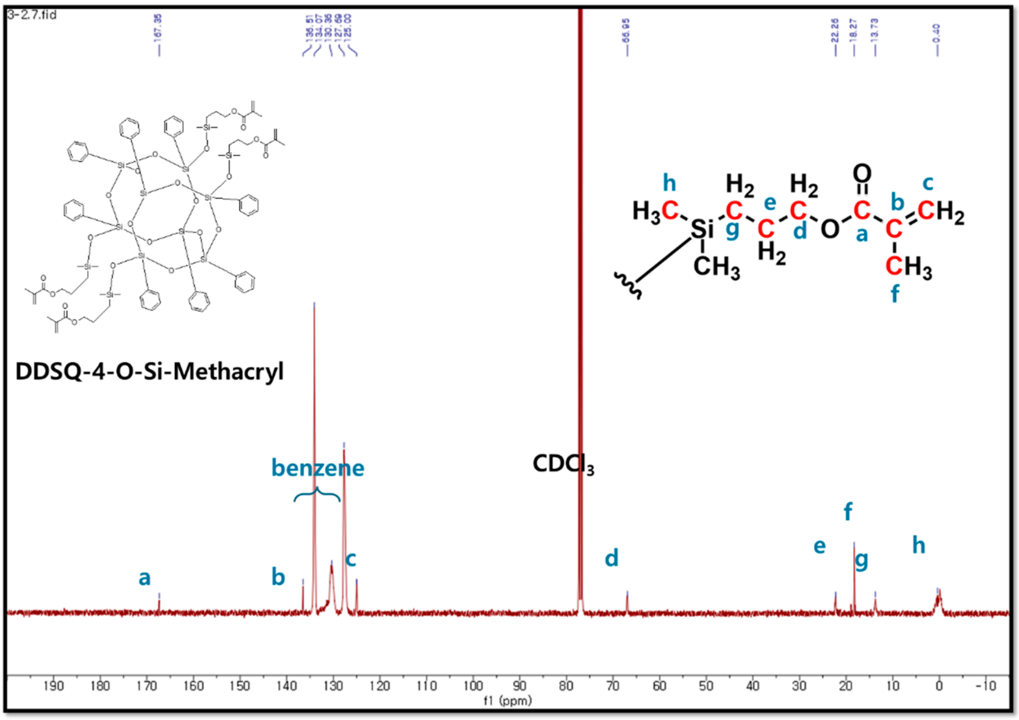

Figure S8. ^{13}C NMR spectrum of DDSQ-4-O-Si-Methacryl (2).

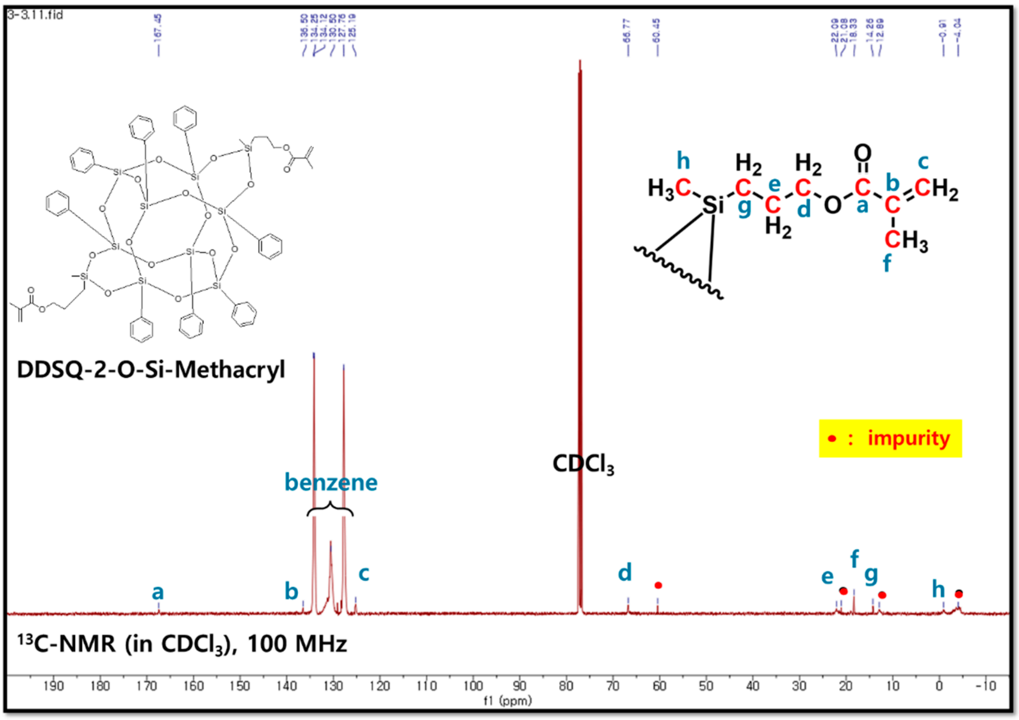

Figure S9. ^{13}C NMR spectrum of DDSQ-2-O-Si-Methacryl (3).

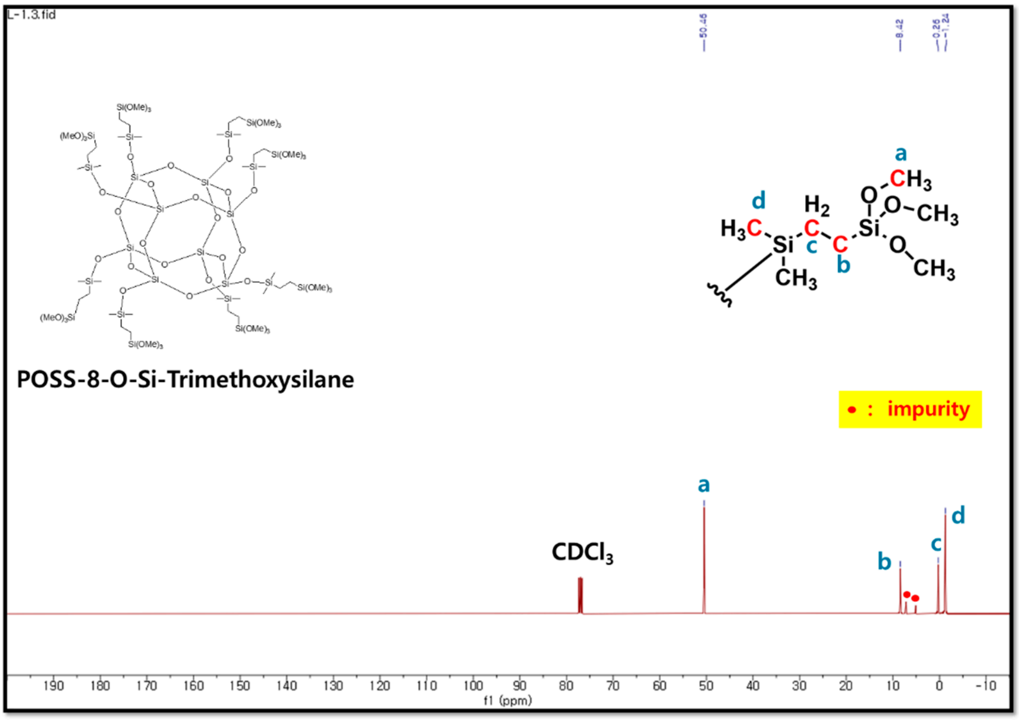

Figure S10. ^{13}C NMR spectrum of POSS-8-O-Si-Trimethoxysilane (4).

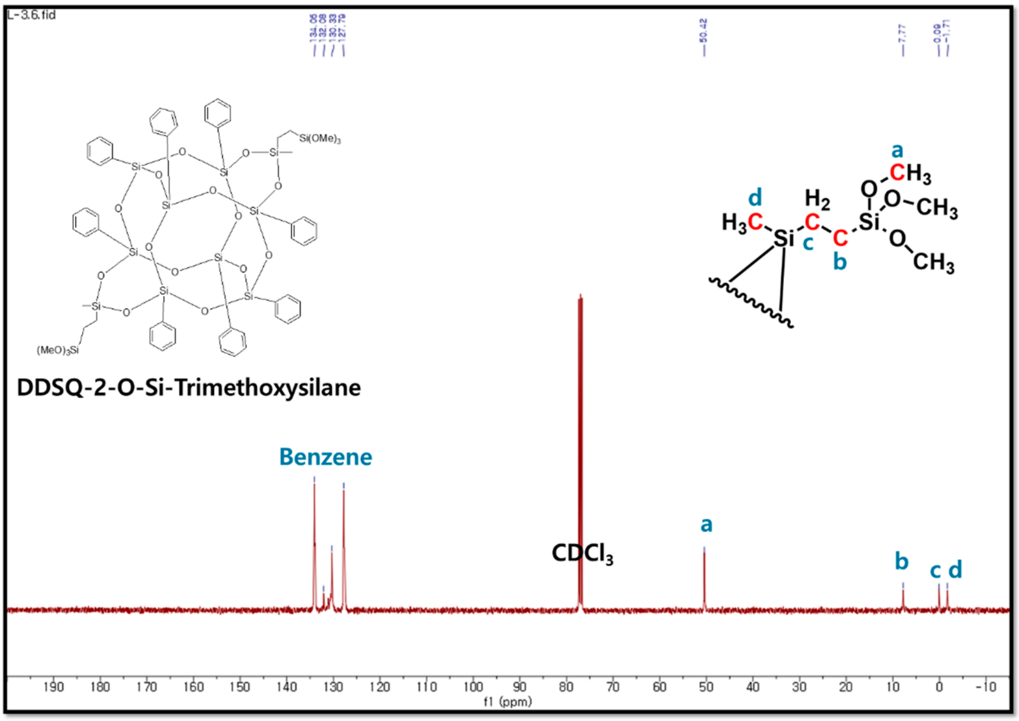

Figure S11. ^{13}C NMR spectrum of DDSQ-4-O-Si-Trimethoxysilane (5).

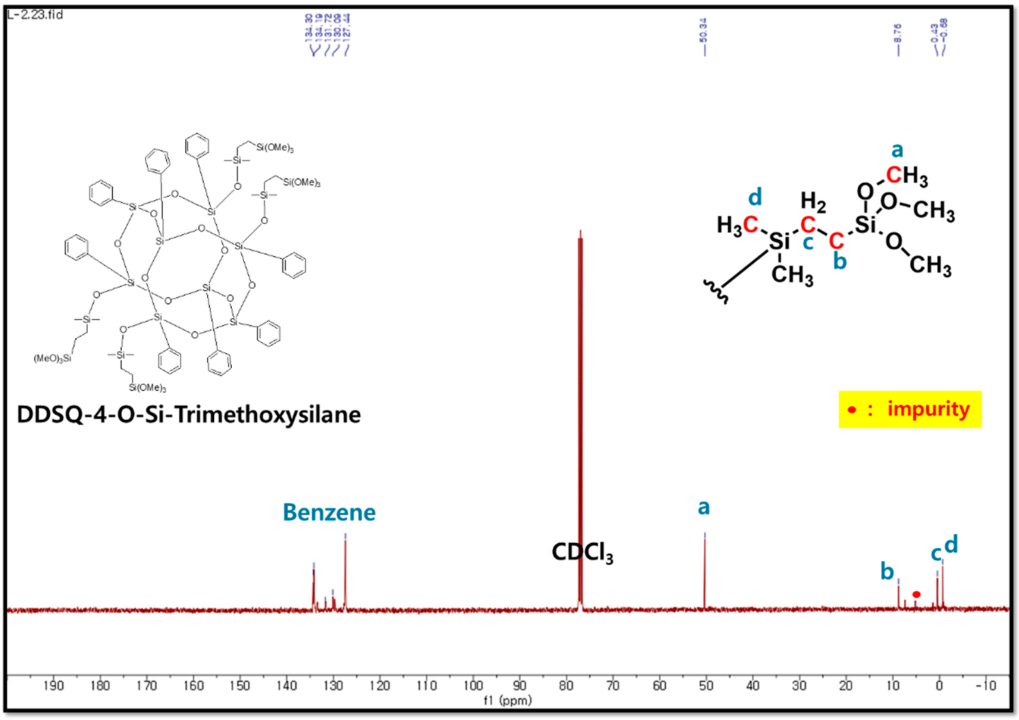

Figure S12. ^{13}C NMR spectrum of DDSQ-2-O-Si-Trimethoxysilane (6).

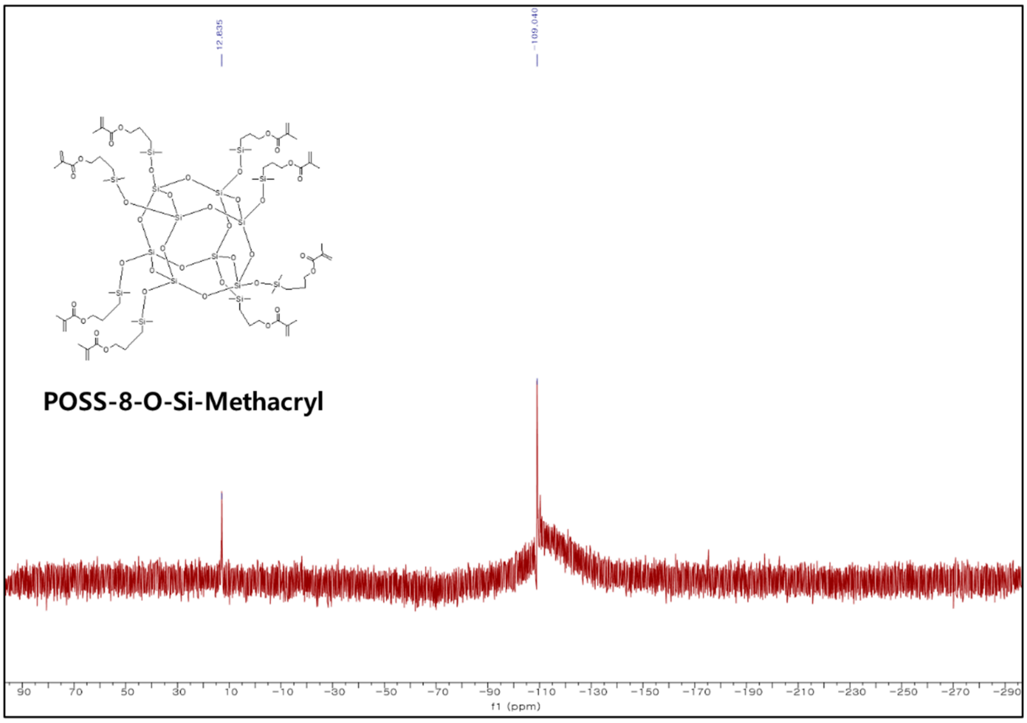

Figure S13. ^{29}Si NMR spectrum of POSS-8-O-Si-Methacryl (1).

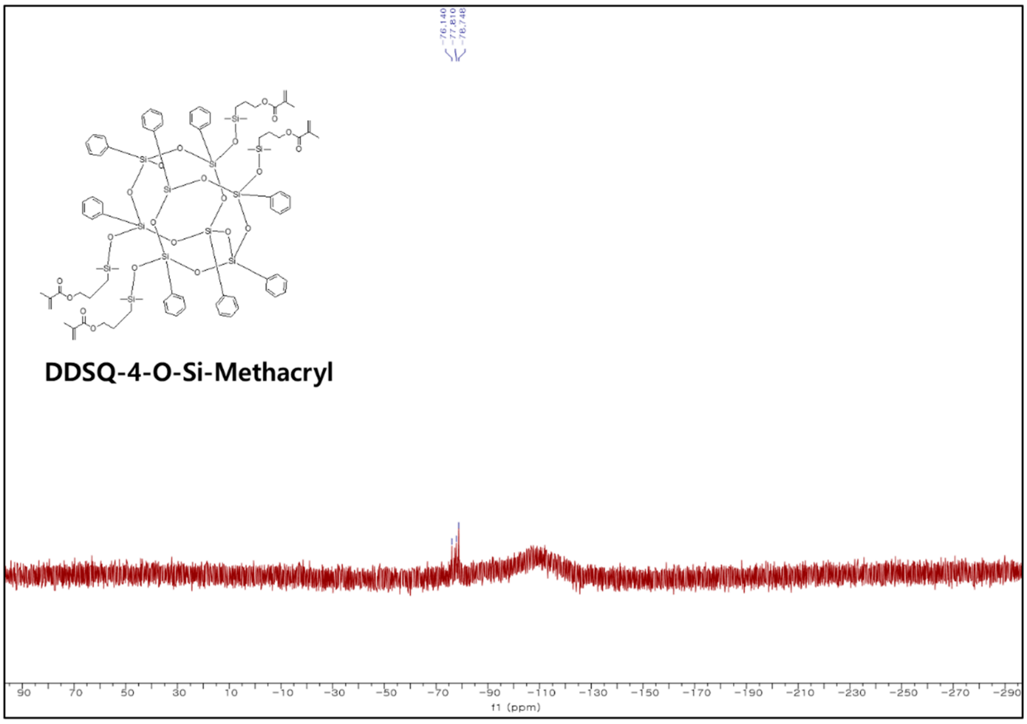

Figure S14. ^{29}Si NMR spectrum of DDSQ-4-O-Si-Methacryl (2).

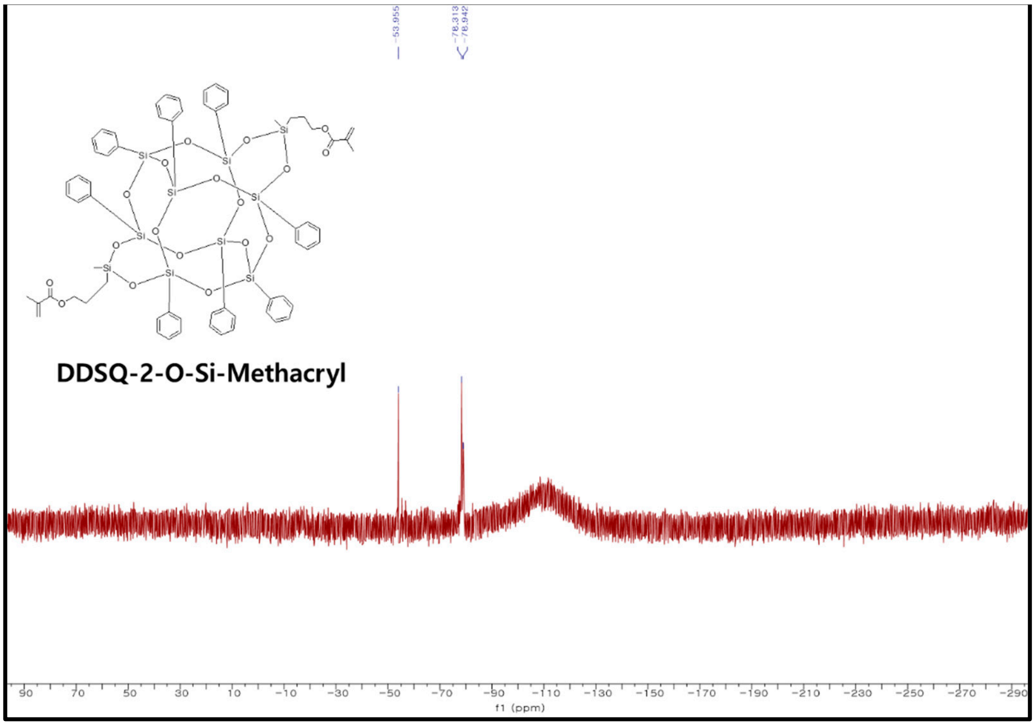

Figure S15. ^{29}Si NMR spectrum of DDSQ-2-O-Si-Methacryl (3).

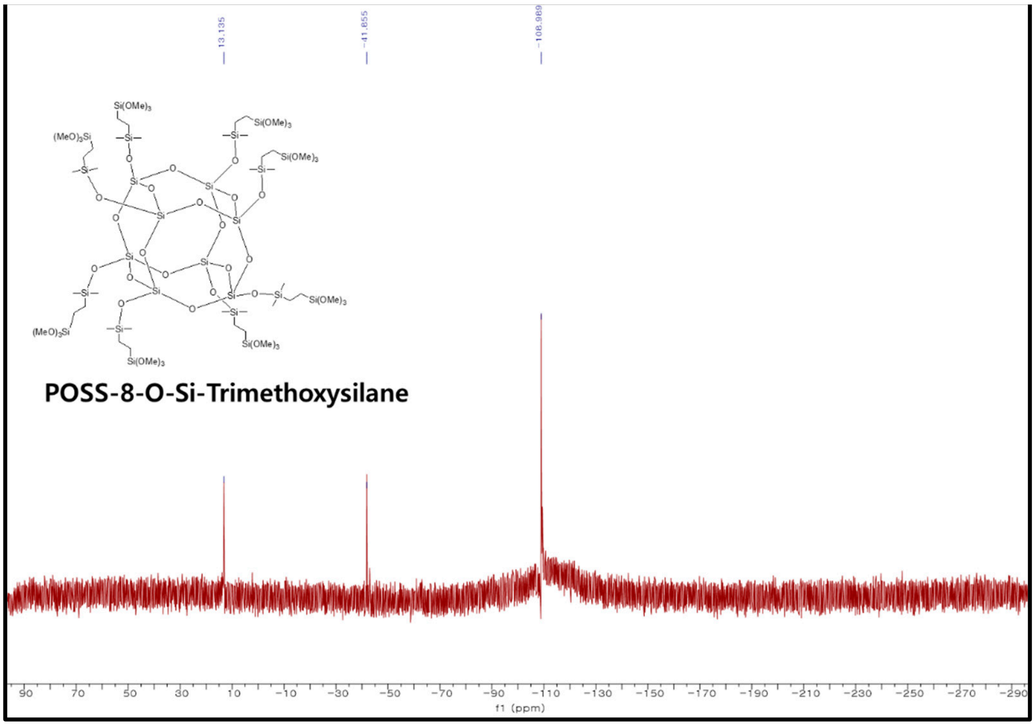

Figure S16. ^{29}Si NMR spectrum of POSS-8-O-Si-Trimethoxysilane (4).

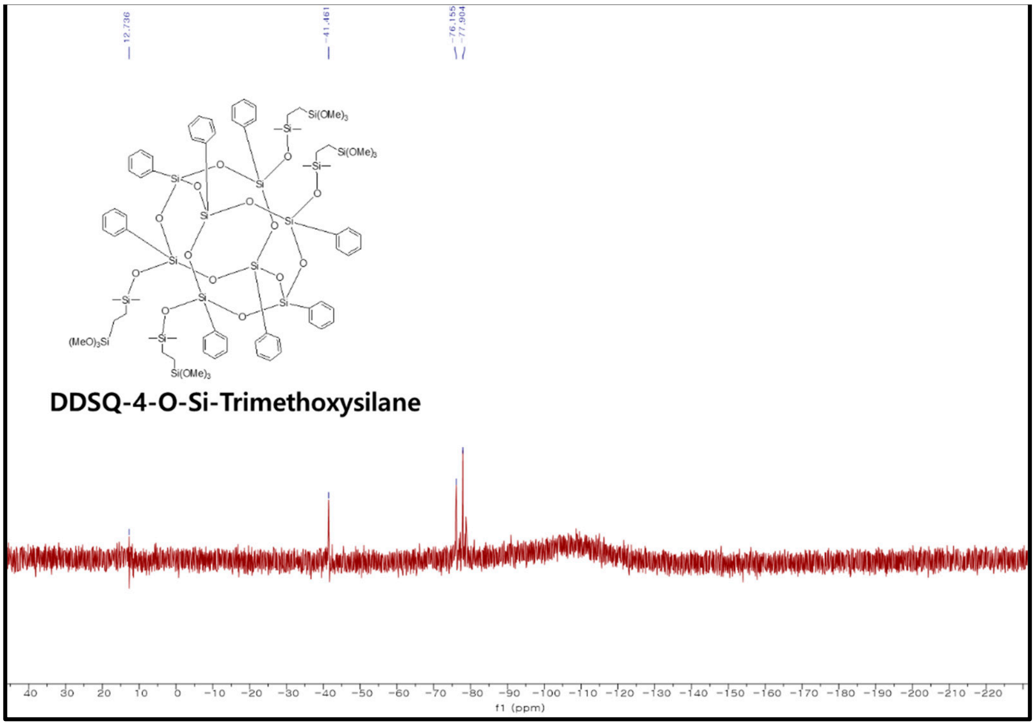

Figure S17. ^{29}Si NMR spectrum of DDSQ-4-O-Si-Trimethoxysilane (5).

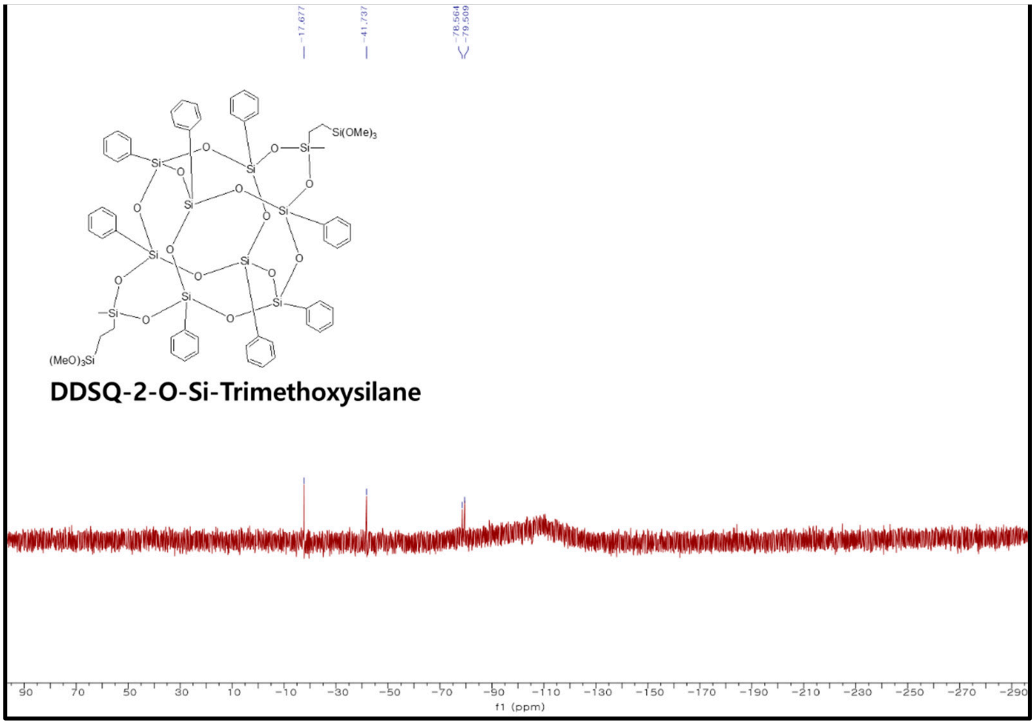

Figure S18. ^{29}Si NMR spectrum of DDSQ-2-O-Si-Trimethoxysilane (6).

Mass Spectroscopy

Mass spectrometric analysis was performed using matrix-assisted laser desorption/ionization time-of-flight mass spectrometry (MALDI-TOF MS). Sodium acetate (NaOAc) was used as a cationization agent, and super-DHB was employed as the matrix. The synthesized compound was dissolved in tetrahydrofuran (THF) at a concentration of 5 mg mL^{-1}. The sample solution, matrix solution, and NaOAc solution were mixed at a volume ratio of 50:50:1 prior to analysis.

MALDI-TOF MS measurements were conducted in positive-ion mode. Since Na^+ was used to promote ionization of the analyte, the molecular ion was predominantly detected as the sodium adduct, $[M + Na]^+$, rather than as the neutral molecular species.

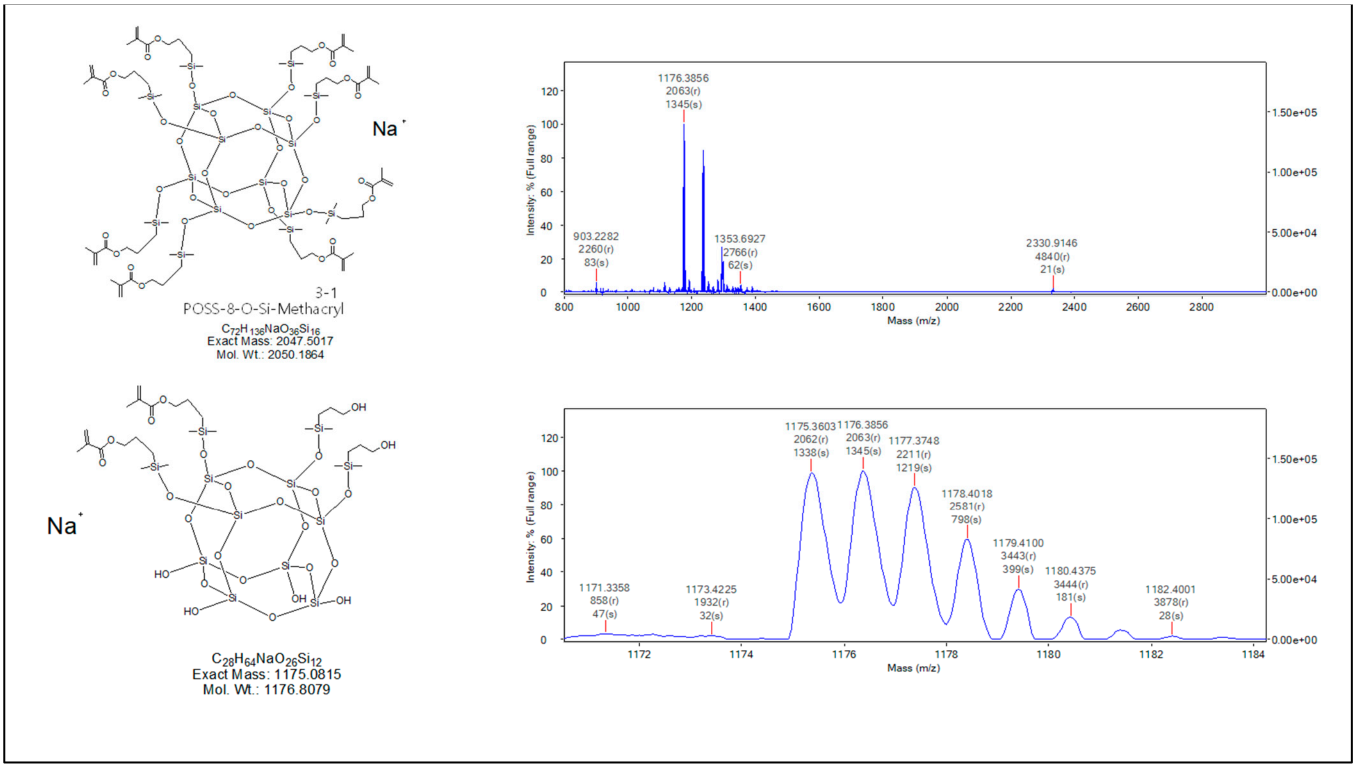

Figure S19. MALDI-TOF Mass spectrum of POSS-8-O-Si-Methacryl (1).

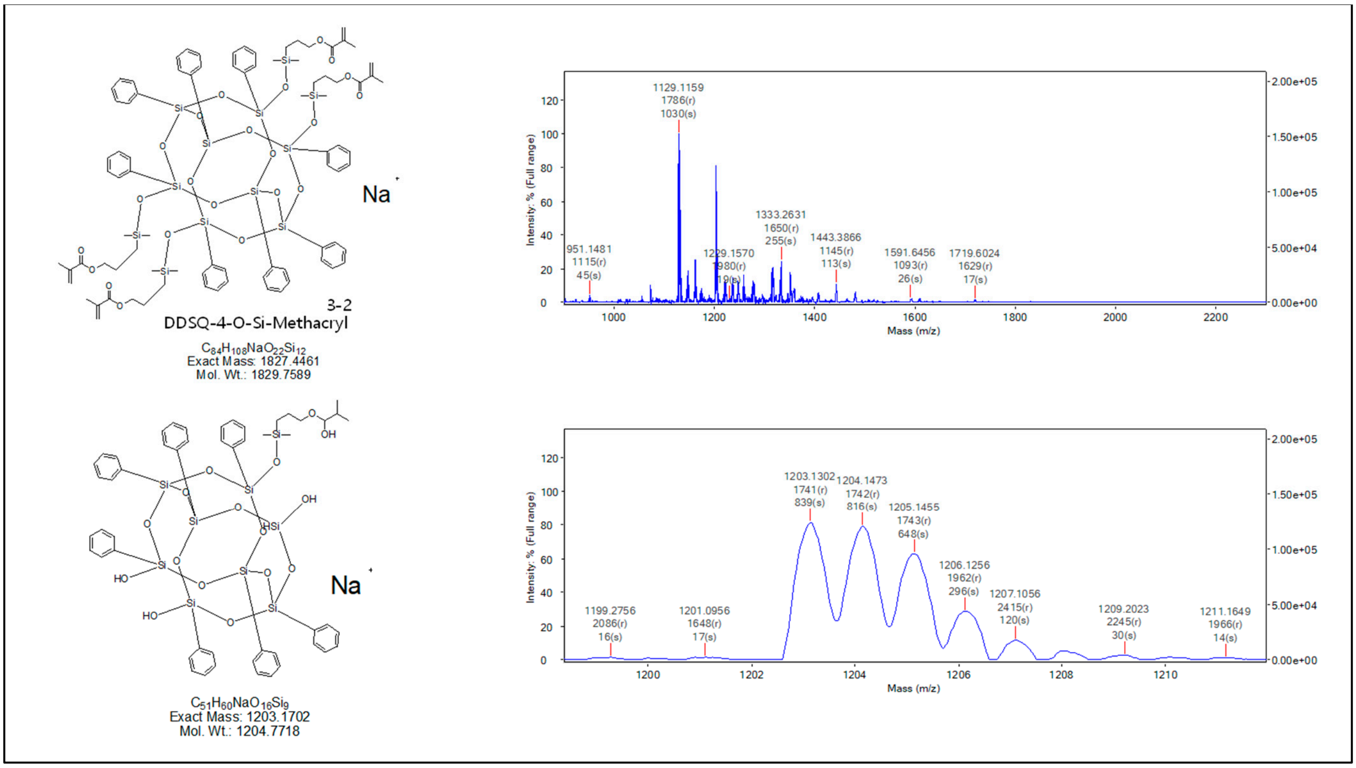

Figure S20. MALDI-TOF Mass spectrum of DDSQ-4-O-Si-Methacryl (2).

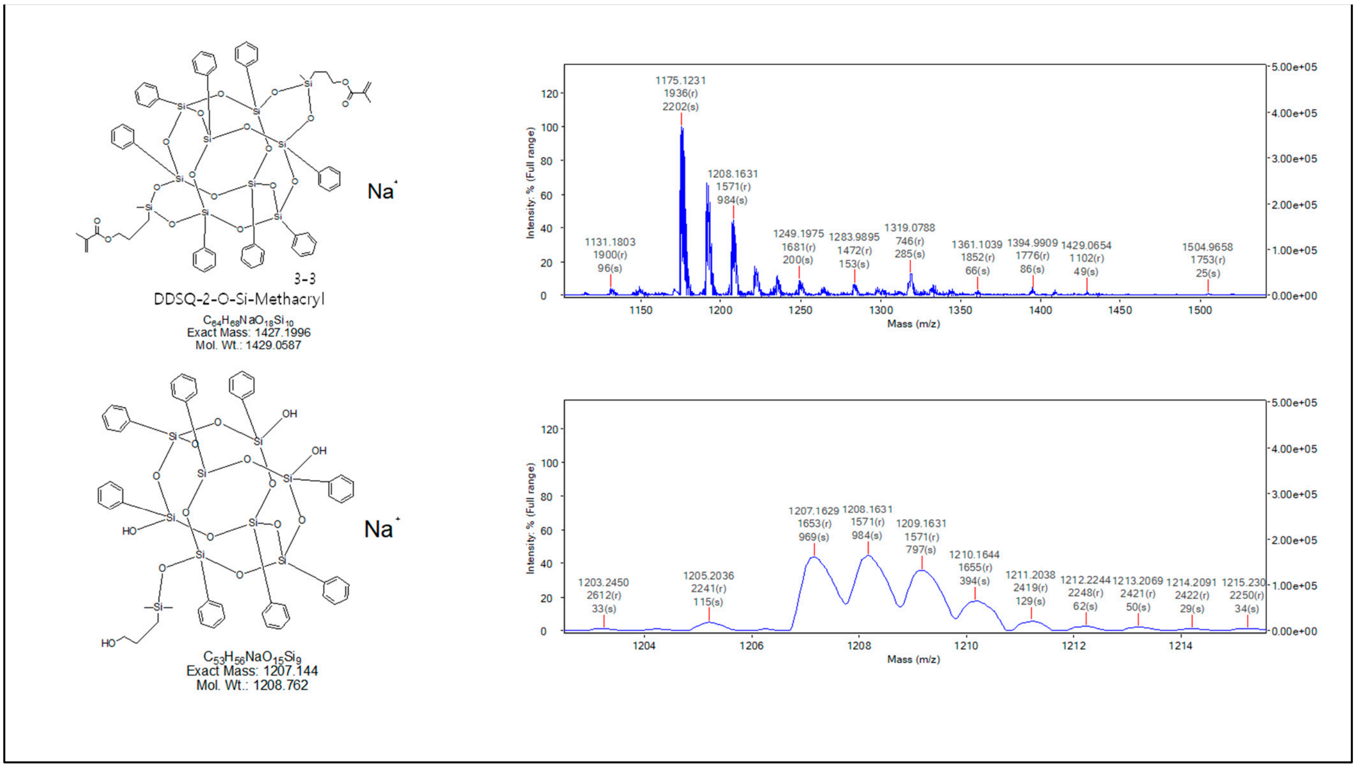

Figure S21. MALDI-TOF Mass spectrum of DDSQ-2-O-Si-Methacryl (3).

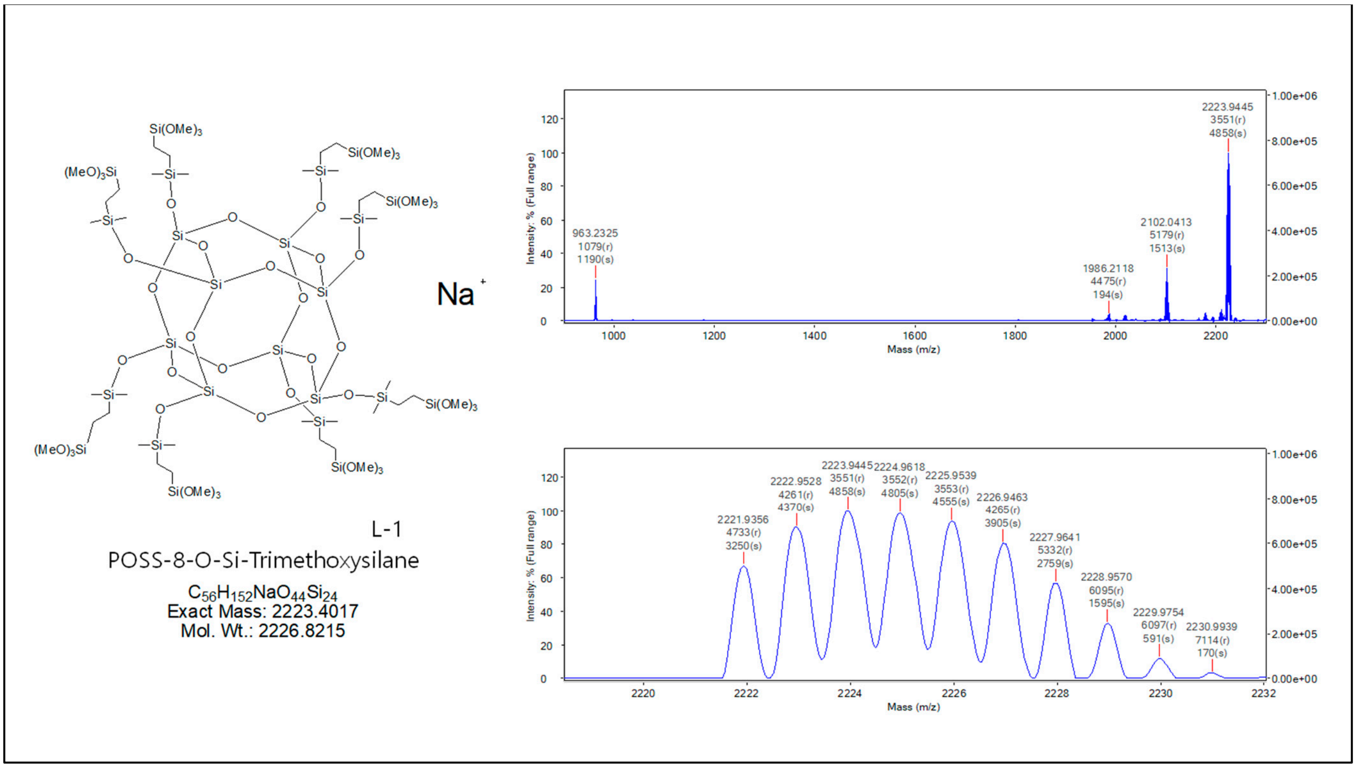

Figure S22. MALDI-TOF Mass spectrum of POSS-8-O-Si-Trimethoxysilane (4).

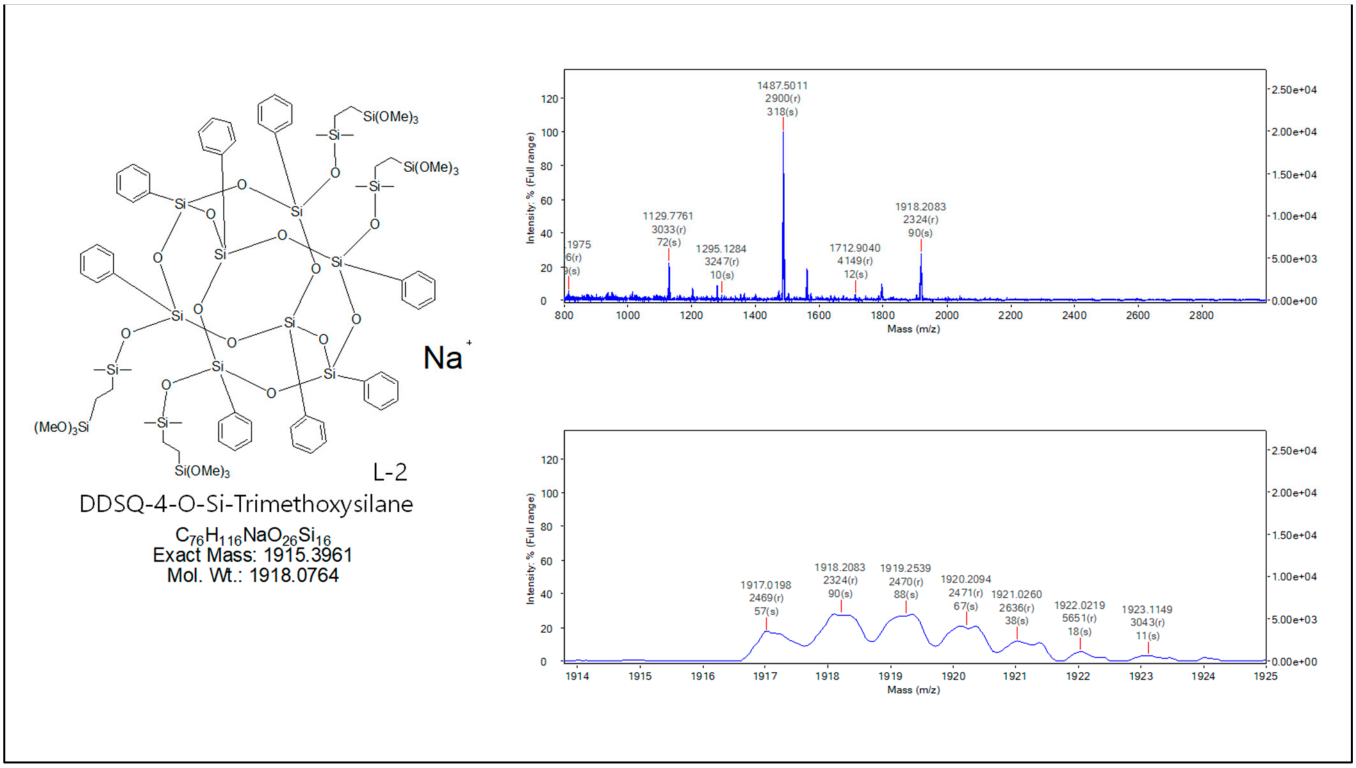

Figure S23. MALDI-TOF Mass spectrum of DDSQ-4-O-Si-Trimethoxysilane (5).

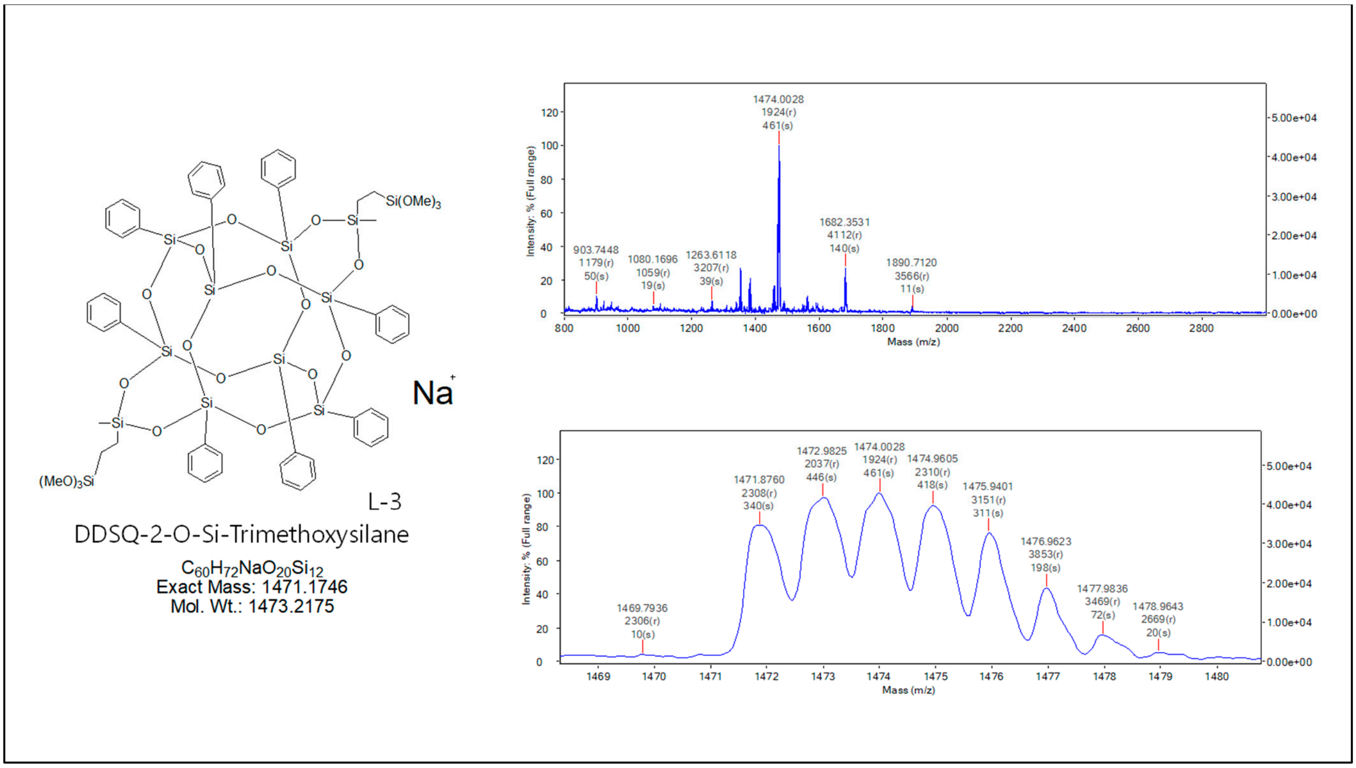

Figure S24. MALDI-TOF Mass spectrum of DDSQ-2-O-Si-Trimethoxysilane (6).

VI. References

1. Jesberger, M.; Barner, L.; Stenzel, M.H.; Malmström, E.; Davis, T.P.; Barner-Kowollik, C. Hyperbranched polymers as scaffolds for multifunctional reversible addition–fragmentation chain-transfer agents: A route to polystyrene-*core*-polyesters and polystyrene-*block*-poly(butyl acrylate)-*core*-polyesters. *J. Polym. Sci. Part A Polym. Chem.* **2002**, *40*, 3847–3861, https://doi.org/10.1002/pola.10976.
2. Provatas, A.; Luft, M.; Mu, J.C.; White, A.H.; Matisons, J.G.; Skelton, B.W. Silsesquioxanes: Part I: A key intermediate in the building of molecular composite materials. *J. Organomet. Chem.* **1998**, *565*, 159–164, https://doi.org/10.1016/s0022-328x(98)00450-1.
3. Hoque, M.A.; Kawakami, Y. Synthesis of Polysilsesquioxanes with Double-Decker Silsesquioxane Repeating Units. *J. Sci. Res.* **2016**, *8*, 217–227, https://doi.org/10.3329/jsr.v8i2.26791.
4. Hasegawa, I.; Sakka, S.; Sugahara, Y.; Kuroda, K.; Kato, C. Silicate anions formed in tetramethylammonium silicate methanolic solutions as studied by 29Si nuclear magnetic resonance. *J. Chem. Soc. Chem. Commun.* **1989**, *25*, 208–210, https://doi.org/10.1039/c39890000208.
5. Mituła, K.; Duszczak, J.; Brząkalski, D.; Dudziec, B.; Kubicki, M.; Marciniec, B. Tetra-functional double-decker silsesquioxanes as anchors for reactive functional groups and potential synthons for hybrid materials. *Chem. Commun.* **2017**, *53*, 10370–10373, https://doi.org/10.1039/c7cc03958a.
6. Prządka, D.; Marcinkowska, A.; Andrzejewska, E. POSS-modified UV-curable coatings with improved scratch hardness and hydrophobicity. *Prog. Org. Coat.* **2016**, *100*, 165–172, https://doi.org/10.1016/j.porgcoat.2016.01.011.
7. Władyczyn, A.; Simiczyjew, A.; Nowak, D.; Wądzyńska, J.; John, Ł. Novel hybrid composites based on double-decker silsesquioxanes functionalized by methacrylate derivatives and polyvinyl alcohol as potential materials utilized in biomedical applications. *Biomater. Adv.* **2023**, *146*, 213290, https://doi.org/10.1016/j.bioadv.2023.213290.
8. Sztorch, B.; Brząkalski, D.; Głowacka, J.; Pakuła, D.; Frydrych, M.; Przekop, R.E. Trimming flow, plasticity, and mechanical properties by cubic silsesquioxane chemistry. *Sci. Rep.* **2023**, *13*, 14156, https://doi.org/10.1038/s41598-023-40784-4.
